# The dynamic effect of genetic variation on the in vivo ER stress transcriptional response in different tissues

**DOI:** 10.1093/g3journal/jkac104

**Published:** 2022-04-29

**Authors:** Nikki D Russell, Clement Y Chow

**Affiliations:** Department of Human Genetics, University of Utah School of Medicine, Salt Lake City, UT 84112, USA

**Keywords:** ER stress, regulatory variation, G×E, tissue effects, genetic variation, in vivo mouse

## Abstract

The genetic regulation of gene expression varies greatly across tissue-type and individuals and can be strongly influenced by the environment. Many variants, under healthy control conditions, may be silent or even have the opposite effect under diseased stress conditions. This study uses an in vivo mouse model to investigate how the effect of genetic variation changes with cellular stress across different tissues. Endoplasmic reticulum stress occurs when misfolded proteins accumulate in the endoplasmic reticulum. This triggers the unfolded protein response, a large transcriptional response which attempts to restore homeostasis. This transcriptional response, despite being a conserved, basic cellular process, is highly variable across different genetic backgrounds, making it an ideal system to study the dynamic effects of genetic variation. In this study, we sought to better understand how genetic variation alters expression across tissues, in the presence and absence of endoplasmic reticulum stress. The use of different mouse strains and their F1s allow us to also identify context-specific *cis-* and *trans-* regulatory variation underlying variable transcriptional responses. We found hundreds of genes that respond to endoplasmic reticulum stress in a tissue- and/or genotype-dependent manner. The majority of the regulatory effects we identified were acting in *cis-*, which in turn, contribute to the variable endoplasmic reticulum stress- and tissue-specific transcriptional response. This study demonstrates the need for incorporating environmental stressors across multiple different tissues in future studies to better elucidate the effect of any particular genetic factor in basic biological pathways, like the endoplasmic reticulum stress response.

## Introduction 

Genetic variation rarely acts in isolation and changes in environmental conditions or tissue-type can drastically alter the effect of a particular variant (Eichler *et al.* 2010; Nickels *et al.* 2013; Matsui and Ehrenreich 2016; Rask-Andersen *et al.* 2017). This genotype × environment (G×E) effect is pervasive in biology and is defined as a genotype-specific phenotypic response to different environments (Grishkevich and Yanai 2013). G×E effects are particularly evident in RNA levels where context-specific changes in expression are the norm (Li *et al.* 2006; Hodgins-Davis and Townsend 2009; Grishkevich *et al.* 2012; Glass *et al.* 2013). Tissue-type can have significant effects on gene expression across different genetic backgrounds. For example, the GTEx consortium has made significant progress in understanding the genetic architecture underlying gene expression across tissues in healthy human individuals and the large effect that different tissues can have on these patterns (GTEx Consortium 2017). By studying G×E effects in different tissues, we are uncovering tissue-specific genetic mechanisms that underly a variable phenotypic response to environmental stimuli. Without including this complexity, we risk missing important elements of these interactions. This study uses the endoplasmic reticulum (ER) stress response to evaluate how G×E interactions alter gene expression across tissue type and stress conditions.

The ER is a major site of protein and lipid synthesis, protein folding, and calcium storage (Alberts *et al.* 2002). ER stress occurs when misfolded proteins accumulate in the ER lumen because of overwhelming protein folding demands or improper protein folding (Lin *et al.* 2008). Cells respond to ER stress with the well-conserved unfolded protein response (UPR), a coordinated series of cellular processes that reduces ER protein load and increases the ability of the ER to clear misfolded proteins (Travers *et al.* 2000; Jonikas *et al.* 2009). The UPR consists of changes in gene expression which is initiated through the 3 main signaling branches of the UPR: IRE1, ATF6, and PERK (Ron and Walter 2007). If the UPR is unable to restore ER homeostasis, it will activate apoptosis pathways. The response to misfolded proteins is essential for maintenance of basal cellular conditions and critical when there are any changes in the cellular environment (Ron and Walter 2007).

Despite being a conserved, basic cellular function, the ER stress response is subject to inter-individual variation in *Drosophila*, mouse, and humans (Dombroski *et al.* 2010; Chow *et al.* 2013, 2015, 2016). In *Drosophila*, we used natural genetic variation to demonstrate that susceptibility to ER stress is highly variable across strains and is associated with single nucleotide polymorphisms (SNPs) in canonical and novel ER stress genes (2013, Chow *et al.* 2016). We also used mouse embryonic fibroblasts (MEFs) from inbred mouse strains to uncover a complex *cis-* and *trans-* genetic architecture underlying the variable transcriptional response to ER stress among different genetic backgrounds (Chow *et al.* 2015). Another study demonstrated that the ER stress response is variable among immortalized human B cells from diverse individuals (Dombroski *et al.* 2010). These previous studies show that there is an entire layer of genetic variation that is silent under healthy conditions, but alters expression under ER stress. However, all past mouse and human studies examining the G×E interactions under ER stress utilized in vitro cell culture. G×E studies in other contexts have clearly shown that genetic architecture changes drastically across different tissue types (GTEx Consortium 2017; Taylor *et al.* 2018; Marderstein *et al.* 2021).

Here, we report that tissue type and ER stress have strong effects on how genetic variation impacts transcript levels. We identified hundreds of genes that showed variability in their response to ER stress that were dependent on tissue type and genetic background. These genes are enriched for processes, such as metabolism, inflammation, and immunity. Strikingly, in contrast to previous studies where noncanonical ER stress genes were found to be variable (Chow *et al.* 2015), we found that some genotype-dependent ER stress response genes in vivo are involved in pathways with clear roles in the ER stress response, indicating that at least some variability in response is derived from canonical ER stress genes. Our study design employed F1 mouse crosses to uncover the *cis-* and *trans-* regulatory variation that underlies the variable ER stress transcriptional response. We found that genetic variation has a complex and context-specific role in regulating the variable transcriptional response in the mouse, especially when it comes to tissue type. This study expands the understanding of the ER stress transcriptional response and the *cis-/trans-* regulatory variation that impacts this network in different environmental contexts. Together, these findings have implications for identifying ER stress response modifiers, tissue-specific effects, and elements that make up the highly variable ER stress response.

## Materials and methods

### Mice

C57BL/6J (B6) and CAST/EiJ (CAST) mice were obtained from Jackson Laboratories (Bar Harbor, ME). F1s were generated by crossing female B6 mice to male CAST mice. 6 male mice of each genotype (B6, CAST, and F1) of approximately 15–23 weeks of age were used for the experiment. Mice from each genotype were randomly assigned control or TM treatments. The 3 different genotypes were all housed together based on litters and house separately after treatment. All experiments involving mice were performed according to institutional IACUC and NIH guidelines.

### Tunicamycin injection and RNA extraction

We administered tunicamycin (TM) or DMSO (control) (Sigma) with an intraperitoneal injection. TM induces ER stress by inhibiting protein N-glycosylation in the ER, causing an accumulation of misfolded proteins and a strong UPR (Tkacz and Lampen 1975). TM was dissolved in DMSO (Sigma) to achieve a 2.5 × 10^−4 ^mg/µl concentration. To induce ER stress in the mouse model, we injected mice with a final concentration of 1 mg of TM per 1 kg mouse weight (same concentration for DMSO control mice) (Choy *et al.* 2017; Feng *et al.* 2017). The final concentration of DMSO in the control injection was well below the maximum amount that has been reported to be well tolerated in mice through IP injection (Gad *et al.* 2006). After injection, mice were allowed to recover for 8 h. Mice were then euthanized and organs were harvested and stored at −80°C. RNA was isolated by Trizol (Ambion) and Direct-zol RNA MiniPrep Kit (Zymo Research) RNA column extraction protocol.

### Illumina mRNA sequencing and alignment

mRNA sequencing was performed on 18 samples (3 genotypes × 3 replicates × 2 treatments) for liver and 18 samples for kidney, for a total of 36 samples. Samples were prepared and sequenced by the Huntsman Cancer Institute High-Throughput Genomics Core. Library prep was performed using Illumina TruSeq Stranded Total RNA Library Prep Ribo-Zero Gold. The 36 samples were then sequenced on the NovaSeq 2 × 50 bp Sequencing, for a total of approximately 25 million paired reads per sample. Fastq files were trimmed by using seqtk v1.2 software. Parental RNA-seq reads were aligned to strain-specific reference genomes using Bowtie2 v2.2.9 software (Langmead and Salzberg 2012). B6 and CAST genomes were obtained from Ensembl (http://ftp.ensembl.org/pub/release-103/fasta/; accessed 2022 May 3). Masked reference genomes were created using bedtools v2.28.0 (Quinlan and Hall 2010). Known CAST variants were replaced with ambiguous N nucleotides in the B6 genome. F1 reads were aligned to masked genomes using STAR v2.6 software, to allow for a more variant aware alignment (Dobin *et al.* 2013). Alignment to the respective parental genomes of B6 and CAST had similar alignment rates (B6: 89.18%; CAST: 86.35%). Alignment of the F1 transcripts to the masked genome led to lower alignment rates, most likely due to the ambiguous nature of the masked genome, but still fell within the acceptable range for RNA-seq analysis (F1: 70.08%) (Conesa *et al.* 2016; Musich *et al.* 2021). F1 alignment to a masked genome does not appear to favor the reference genome (B6) based on our results which showed no bias toward the B6 allele (Supplementary Fig. 1, a and b). Alignment files were sorted and converted using samtools v1.12 (Li *et al.* 2009).

### Quantification of expression levels

The Deseq2 default normalization method (median of ratios) was used to normalize counts (Love *et al.* 2015). For each genotype, condition, and tissue type, principal component analysis was used to identify outlying samples. For a given tissue, within a genotype, we required the TM samples to be clustered together and the control samples to be clustered together. We also performed clustering and heatmap analyses with the gene expression data for each group to look for sample clustering of the TM samples and the control samples. If a given replicate was not with the appropriate cluster in the principle component analysis (PCA) and the sample clustering heatmaps, it was removed from the analysis. At least 2 replicates remained after removing outliers for each combination of tissue, genotype, and condition. The exact number of resulting samples were:

Liver:

B6 control: *N* = 3; TM: *N* = 2

CAST control: *N* = 2; TM: *N* = 3

F1 control: *N* = 3; TM: *N* = 2

Kidney:

B6 control: *N* = 3; TM: *N* = 3

CAST control: *N* = 3; TM: *N* = 3

F1 control: *N* = 3; TM: *N* = 3

Remaining samples were reanalyzed using Deseq2 v1.28.1. A gene was considered “expressed” if the Deseq2 value of base mean was ≥5. A gene was considered significantly altered by ER stress if it met a 1.5-fold (5% FDR) change cutoff. Despite some groups containing lower sample sizes due to outlier removal, we are still confident in our results. Deseq2 is designed to offer consistent performance, even for small studies with few replicates (Love *et al.* 2014).

### Effect of genetic background and tissue type on fold-change levels

To identify genes that were significantly impacted by tissue-type in each individual genotype, we used the same Deseq2 pipeline described above which uses the default Wald-test, but incorporated tissue and condition as interaction terms. Small *P*-values from this study design indicates that the log fold change due to treatment (ER stress) is significantly different for the 2 tissues. *P*-adj values were calculated using the default Deseq2 and multiple testing was accounted for using the Benjamini and Hochberg method. We used a *P*-adjusted cutoff of 0.05. This was performed for each of the 3 genotypes. By incorporating tissue and condition as interaction terms [ddsSE <- DESeqDataSet(SE, design = ∼ tissue + condition + tissue: condition)] we are controlling for any fundamental differences between the tissues that are not affected by ER stress. If a gene were fundamentally different between the 2 tissues but responded to ER stress in a similar manner, this gene would not be identified as significant in our analysis. We are only identifying genes that respond to ER stress differently between the 2 tissues. To assess quality of this model, we created MA plots for each of the 3 genotypes tested, comparing the 2 tissues (Supplementary Fig. 2). There was no dependence or any other anomalous behavior such as batch effects seen.

To identify genes in a given tissues that is impacted by genetic background, we used a likelihood ratio test (LRT) that is provided in the Deseq2 software. An LRT is used to identify genes that show change in fold-change across the 3 different genetic backgrounds after the induction of ER stress. Significant *P*-values indicate a gene that has a change in fold-change, across the 3 genotypes, in any combination, determined solely by the difference in deviance between the full and reduced model formula. We used the default Deseq2 Benjamini and Hochberg method to adjust for multiple tests and calculate our *P*-adjusted values which we filtered on (*P*-adj < 0.05). We used a hypergeometric distribution test to test for overlap of these sets of genes.

### Allele-specific expression quantification in the F1 mouse

Allele-specific expression was quantified using GATK ASEReadcounter v3.8 (McKenna *et al.* 2010). SNP information was obtained from the Sanger Mouse Genomes Project (https://www.sanger.ac.uk/data/mouse-genomes-project/; accessed 2022 May 3). Counts for all replicates were combined to increase coverage and reduce variability. To increase the reliability of counts, we only included genes in our analyses that had at least 2 informative SNPs between B6 and CAST and at least 20 counts in at least one of the conditions for both *cis-/trans-* analysis and for ASE analysis. This resulted in 5,669 genes in liver and 7,764 genes in kidney. Genes were considered to have a significant change in ASE post-ER stress if the ratio of the expression of the 2 alleles (B6 and CAST) under control conditions was significantly different than the ratio of expression under TM conditions determined by the Fisher’s exact test followed by a 5% FDR correction.

### Determination of regulatory effect

We used F1 hybrid mice to quantify allele-specific expression and partition the effects of genetic variation on gene expression into *cis-* and *trans-* effects. Classification of *cis-* and *trans-* effects was performed using previously published methodology (McManus *et al.* 2010; Chow *et al.* 2015). Category naming and classification is consistent with established terminology for this type of study (McManus *et al.* 2010; Chow *et al.* 2015). In order to determine if a gene is impacted by a *cis-* or *trans-* effect, we generated F1 hybrid mice by crossing the highly divergent parental strains; female B6 to male CAST. Transcripts from F1 mice can be assigned to a parental chromosome based on parental SNPs in the spliced transcript. ASE cannot be performed in genes that lack variants in the spliced transcript. *cis-* and *trans-* effects for a particular transcript are assigned by comparing the ratio of allelic expression in the F1 to the ratio of total expression between the parental strains. In the F1 hybrid mouse, both parental alleles are exposed to the same *trans-* factors. Therefore, the ratio of allelic expression is a measure of *cis-* regulatory variation between the 2 parental strains. If the allelic ratio matches the parental expression ratio, the expression difference is attributed to *cis-* regulatory variation. If the allelic ratio differs from the parental expression ratio, the expression difference is attributed to *trans-* regulatory variation.

The requirements for these classifications are highlighted in Supplementary Fig. 3. We used a 0.1% FDR *P*-adj cutoff. To further confirm if a gene that exhibited a regulatory effect did so in only 1 condition, we performed a chi-square test. We recognize that with our lower power due to smaller sample sizes, we are missing some true effects at subthreshold levels. With greater power, we would be capturing more of these true effects. However, we will always miss some true effect due to subthreshold levels. We are confident that we are identifying genes with the most significant levels and most significant impacts on gene expression.

### Enrichment analyses

All gene ontology analyses were performed with DAVID v6.8 (Huang *et al.* 2009). We used the Benjamini-Hochberg method for adjusting for multiple testing. We used adjusted *P*-values to determine significance of enrichment terms and use a *P*-adj. value cutoff of 0.05. Transcription factor (TF) binding site enrichments were identified by using oPOSSUM v3.0 (Ho Sui *et al.* 2005). We used the mouse single site analysis (SSA) tool with a cutoff of 2,000 base pairs up- and downstream of the transcription start site.

## Results

### In vivo ER stress induced by TM

**Fig. 1.**
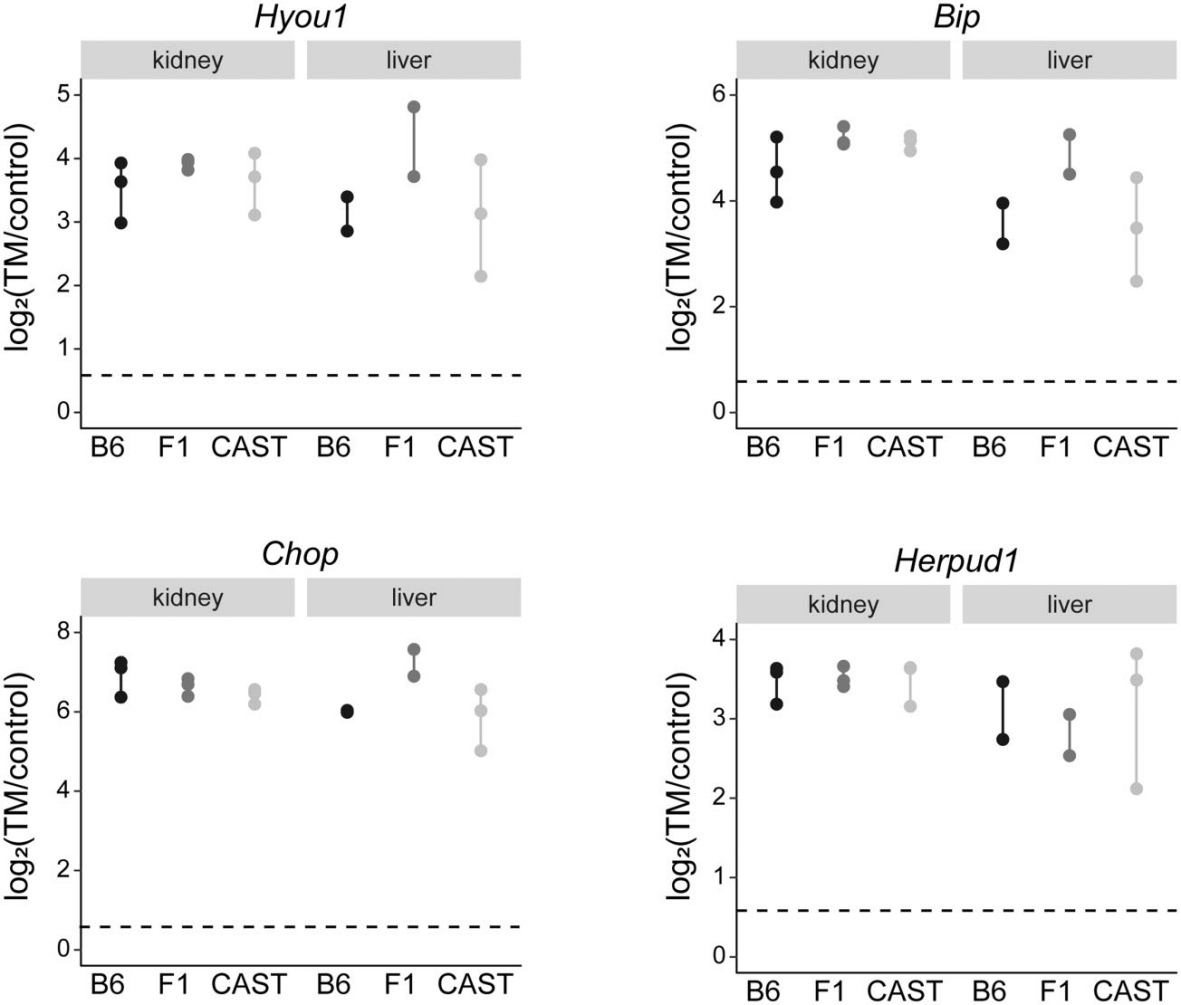
Upregulation of canonical ER stress genes across genotypes and tissues. Log_2_(TM/Control) is plotted for genes with known functions in the ER stress response. Dotted line indicates a 1.5-fold change in gene expression. Each point represents a biological replicate.

To evaluate the extent to which ER stress and tissue type affects gene expression in diverse genetic backgrounds, we induced ER stress in different strains of mice. To induce ER stress, we injected B6 mice, CAST mice, and their F1 hybrid progeny with TM. TM causes ER stress by blocking N-linked glycosylation through the inhibition of DPAGT1, which catalyzes the first step of N-linked glycosylation. TM treatment results in a robust UPR and is a standard tool for inducing ER stress (Tkacz and Lampen 1975; Heifetz *et al.* 1979; Bassik and Kampmann 2011). For this study, we focused on liver and kidney, tissues that rely heavily on protein transport and secretion. Proper ER function and response to ER stress plays a large part in their function. In fact, ER stress and aberrant protein trafficking is pathogenic in a large number of liver and kidney diseases (Schaeffer *et al.* 2014; Liu and Green 2019). A *XBP1* splicing assay and RT-qPCR of *BiP*, a canonical ER stress gene, show that TM injection induced a strong ER stress response (Oslowski and Urano 2011) (Supplementary Fig. 4). To determine the full transcriptional response to TM-induced ER stress, kidney and liver samples were analyzed by RNA-seq. ER stress-induced gene expression changes were identified by comparing control and TM samples in a tissue- and genotype-specific manner (Supplementary Tables 1 and 2). At a cutoff of 1.5-fold (5% FDR) change in transcript level, Gene Ontology (GO) enrichment analyses revealed enrichment for canonical ER stress response genes in all tissues and genotypes (Supplementary Table 3). Many canonical ER stress response genes, including *Hyou1*, *Hspa5* (*BiP*), *Ddit3* (*Chop*), and *Herpud1* (Kozutsumi *et al.* 1988; Wang *et al.* 1996; Kokame *et al.* 2000; Wang, Wu *et al.* 2011; Fig. 1), were significantly upregulated in both tissues and all 3 genotypes, indicating a strong UPR. As expected, injection of TM induces a robust in vivo ER stress-response.

Genes that were upregulated in response to ER stress regardless of genotype or tissue-type were enriched for binding sites of known UPR TFs (Supplementary Tables 1 and 4). One of these TFs is NFYA (*z* = 54.8), which binds in conjunction with ATF6, to 2 sites known as the ER Stress Response Elements I and II (ERSEI and ERSEII) (Yoshida *et al.* 2000; Yamamoto *et al.* 2004). There was also enrichment for CEBPA binding sites (Z-score = 3.4), which can bind in conjunction with CHOP (Chikka *et al.* 2013), a downstream component of the UPR involved in ER stress induced apoptosis signaling (Nishitoh 2012).

### ER stress-induced fold-change in different tissues

**Fig. 2.**
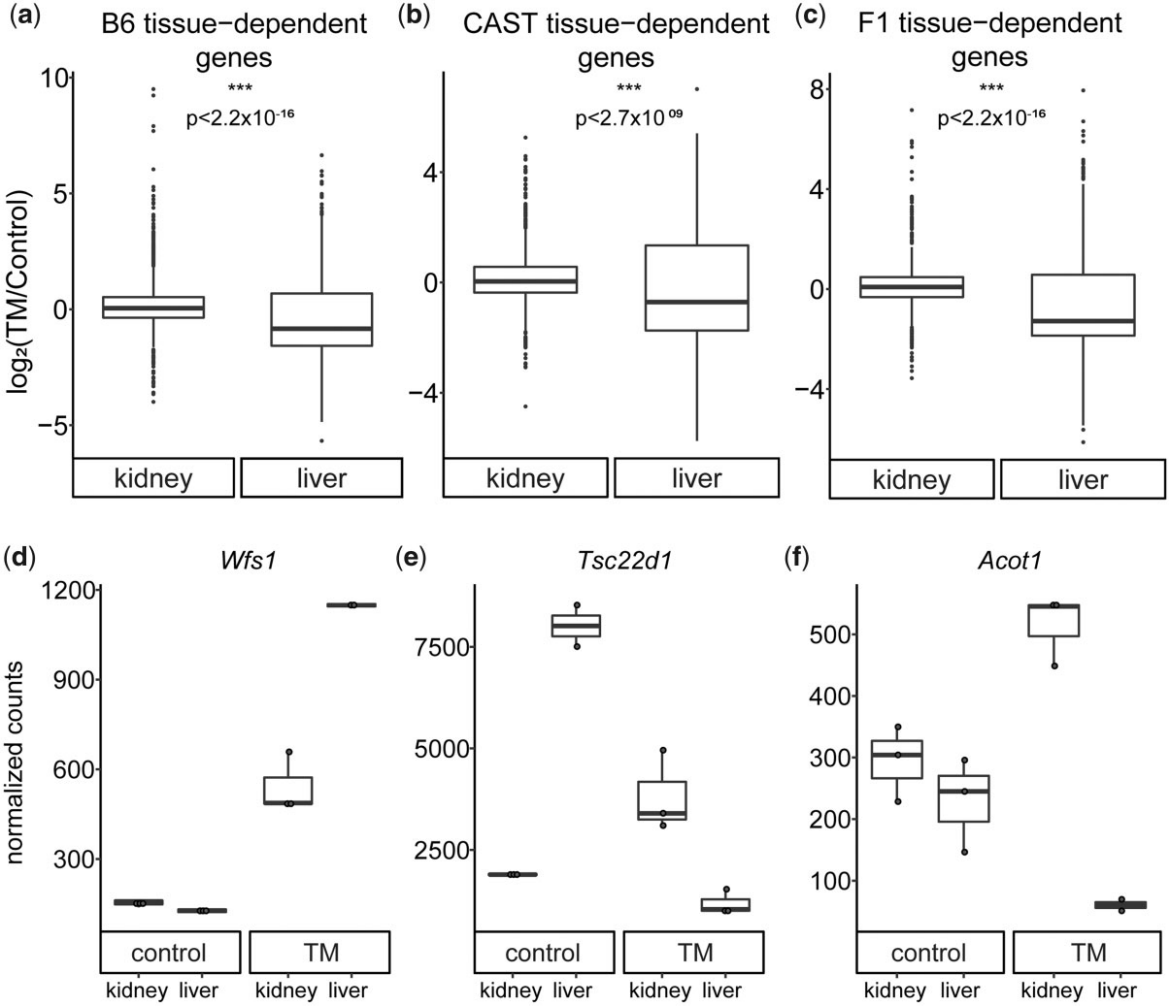
Tissue-specific expression post ER stress response. Log2(TM/Control) is plotted for each gene that displays a significant tissue-specific response to ER stress in B6 (a), CAST (b), and F1 (c). An ANOVA test was used to test for a tissue effect on expression change. Normalized counts are plotted for genes that display expression with a tissue-effect post ER stress in B6 (d), CAST (e), and F1 (f). Each point represents a biological replicate. All plots show a significant tissue effect (*P*-adj < 0.05).

We first characterized how gene expression is affected by ER stress in liver and kidney in each strain independently. In general, we found that many genes exhibit tissue-specific ER stress responses (B6: 34%; CAST: 21%; F1: 24%). The specifics of each genotype are described below. We first compared the magnitude of the fold change of all genes that display a significant tissue-specific response to ER stress. In all 3 genotypes, the response genes in the liver show a significantly lower mean fold change compared to kidney (B6: Kidney: 0.16; Liver: −0.45; *P* < 2.2 × 10^−16^; CAST: Kidney: 0.17; Liver: −0.26; *P* = 2.67 × 10^−09^; F1: Kidney: 0.15; Liver: −0.62; *P* < 2.2 × 10^−16^) (Fig. 2, a–c).

In B6, there were 3,071 (1,310 upregulated; 1,761 downregulated) and 3,433 genes (1,944 upregulated; 1,489 downregulated) that showed a significant change in expression post ER stress, in liver and kidney, respectively (Supplementary Tables 1 and 2). As expected, in both tissues, pathways like the ER UPR (GO:0030968; liver: *q* = 6.5 × 10^−09^; kidney: *q* = 6.6 × 10^−12^) were enriched. We next compared genes and pathways uniquely affected in each tissue when experiencing ER stress. Our analysis takes tissue-type into account, by including it as an interaction term, when analyzing fold-changes post-ER stress. This eliminates genes that are fundamentally different in both tissues but only affected by TM in one. Thus, our analysis requires a gene to be responding to ER stress in both tissues and at significantly different levels. Of the genes affected by ER stress in either tissue in B6, 1,751/5,077 genes (34%) showed ER stress-induced expression that differed between the 2 tissues (cutoff of *q* < 0.05) (Supplementary Table 5) (Supplementary Fig. 5a). More than a third of the response genes in B6 show tissue effects emphasizing the tissue-specificity of this response. Four of the top 6 functional enrichment terms for the tissue-effect genes all relate to metabolism (lipid metabolism, GO: 0006629, *q* = 3.7 × 10^−19^; fatty acid metabolism, GO: 0006631, *q* = 3.9 × 10^−08^; metabolic process, GO: 0008152, *q* = 5.2 × 10^−06^; cholesterol metabolic process, GO: 0008203, *q* = 2.2 × 10^−04^) (Supplementary Table 6), indicating a tissue-specific metabolic response to ER stress. Functional enrichment of genes whose ER stress response was not dependent on tissue type reveals many canonical ER stress categories such as ER UPR (GO: 0030968, *q* = 4.0 × 10^−06^), Golgi to ER retrograde vesicle-mediated transport (GO: 0006890, *q* = 1.1 × 10^−04^), and ER-associated ubiquitin-dependent protein catabolic process (GO: 0030433, *q* = 2.4 × 10^−03^) (Supplementary Table 7).

Next, we quantified how tissue type impacts the transcriptional response to ER stress in the distantly related CAST strain. In CAST, there were 3,749 (1,900 upregulated; 1,849 downregulated) and 2,841 genes (1,738 upregulated; 1,103 downregulated) in liver and kidney, respectively, that showed an ER stress-induced change in expression (Supplementary Tables 1 and 2). As in B6, pathways related to the ER stress response (GO:0030968; liver: *q* = 1.5 × 10^−08^; kidney: *q* = 2.4 × 10^−13^) were significantly enriched in both tissues. Of the 5,115 genes affected in either tissue, 1,054 genes showed tissue-dependent responses to ER stress (1,054/5,115; 21%) (Supplementary Table 5) (Supplementary Fig. 5b). Functional enrichment revealed 7 significant categories, 3 of these relate to metabolism, similar to what was observed with B6, again indicating a tissue-specific metabolic response after ER stress induction (lipid metabolic process, GO: 0006629, *q* = 1.4 × 10^−03^; fatty acid metabolic process, GO: 0006631, *q* = 2.2 × 10^−03^; metabolic process, GO: 0008152, *q* = 3.9 × 10^−03^) (Supplementary Table 6). Functional enrichment of tissue-independent genes reveals enrichment for ER stress terms (ER UPR, GO: 0030968, *q* = 3.9 × 10^−08^; response to unfolded protein, GO: 0006986, *q* = 9.4 × 10^−07^) (Supplementary Table 7).

Finally, we asked how tissue type impacts gene expression levels in response to ER stress in the B6/CAST F1 hybrid mouse. In the F1, there were 3,056 genes in liver (1,330 upregulated; 1,726 downregulated) and 2,806 genes in kidney (1,936 upregulated; 870 downregulated) that showed a significant change in expression post ER stress (Supplementary Tables 1 and 2). As expected, these genes are enriched for the ER stress response (GO:0030968; liver: *q* = 2.2 × 10^−09^; kidney: *q* = 4.6 × 10^−12^). Of the 4,738 genes affected in either tissue, 1,130 genes showed variable ER stress-induced expression between the 2 tissues in the F1 hybrid (1,130/4,738; 24%) (Supplementary Table 5) (Supplementary Fig. 5c). We observed enrichment patterns similar to B6 and CAST. There was enrichment for metabolism in the tissue-dependent genes (metabolic process, GO: 0008152, *q* = 1.9 × 10^−24^; lipid metabolic process, GO: 0006629, *q* = 2.9 × 10^−17^; fatty acid metabolic process, GO: 0006631, *q* = 1.1 × 10^−14^; glutathione metabolic process, GO: 0006749, *q* = 9.5 × 10^−07^) and enrichment for ER stress (GO: 0030968, *q* = 1.3 × 10^−07^) in the tissue-independent genes (Supplementary Tables 6 and 7).

Many genes affected by ER stress exhibit a tissue-effect (B6: 34%; CAST: 21%; F1: 24%). In many cases, this tissue-effect is observed in only 1 genotype (B6: 964/1751, 55%; CAST: 405/1064, 38%; F1: 444/1130, 39%) (Supplementary Fig. 6a). This genotype-effect is explored further in the next section. When comparing the genes with tissue-specific effects on post ER stress expression, B6 was the only genotype to have significant functional enrichment for the ER stress response (GO: 0034976; *q* = 2.1 × 10^−02^) (Supplementary Table 6), suggesting that B6 may harbor variation that affects how its tissues differentially utilize canonical ER stress response genes. For example, *Wfs1* is involved in calcium homeostasis in the ER and impacts the ER stress response (Yamada *et al.* 2006; Fonseca *et al.* 2010) and has similar control levels in both tissues in B6, but there is a Log_2_FC of 1.8 in kidney and a Log_2_FC of 3.2 in liver (Fig. 2d) post ER stress. *Wfs1* shows no tissue effect in CAST or the F1. Response to nutrients (*q* = 3.2 × 10^−03^) was the only functional enrichment unique to the CAST genotype (Supplementary Table 6). Many of these nutrient-sensing genes are involved in lipid transport, cholesterol transport, and lipid localization. This indicates a CAST- and tissue-specific response to ER stress involving lipid metabolism and localization. One of the top significant genes in CAST with a tissue-effect was *Tsc22d1* (*P*-adj = 6.64 × 10^−24^) which is significantly upregulated in kidney (Log_2_FC = 1.077) and downregulated in liver (Log_2_FC = −2.7) (Fig. 2e). *Tsc22d1* is a TF responsive to TGFβ and is thought to be a tumor suppressor gene (Hömig-Hölzel *et al.* 2011). Functional enrichment in genes with a tissue-effect in the F1 revealed terms such as cellular amino acid biosynthetic process (*q* = 4.6 × 10^−02^), protein homotetramerization (*q* = 8.1 × 10^−03^), and fatty acid beta-oxidation (*q* = 1.8 × 10^−07^) (Supplementary Table 6). One of the top genes with tissue-dependent expression unique to F1 is *Acot1* (*q* = 2.02 × 10^−06^) (Fig. 2f). *Acot1* impacts the lipid composition of the cell, which can alter membrane components (Liu *et al.* 2020). This implicates, similar to CAST, a tissue- and genotype-specific response to ER stress which involves membrane composition.

We found 1,623 genes, common in all 3 genotypes, whose ER stress response was not affected by tissue type (Supplementary Fig. 6b). As expected, functional enrichment of these genes includes many functions directly related to the ER stress response (ER UPR, GO: 0030968, *q* = 1.8 × 10^−09^; response to unfolded protein, GO: 0006986, *q* = 1.3 × 10^−06^) (Supplementary Table 7). The top significant functional category was ribosome biogenesis (GO: 0042254, *q* = 3.2 × 10^−11^) and the top cellular compartment enrichment category was for the nucleolus (GO: 0005730, *q* = 1.6 × 10^−33^). The nucleolus is integral to ribosome biogenesis. The nucleolus has been shown to play an active role in regulating cellular stress, with potential links to the ER stress response (Yang *et al.* 2018; Chen and Stark 2019; Pecoraro *et al.* 2020).

### ER stress-induced fold-change varies by genetic background

**Fig. 3.**
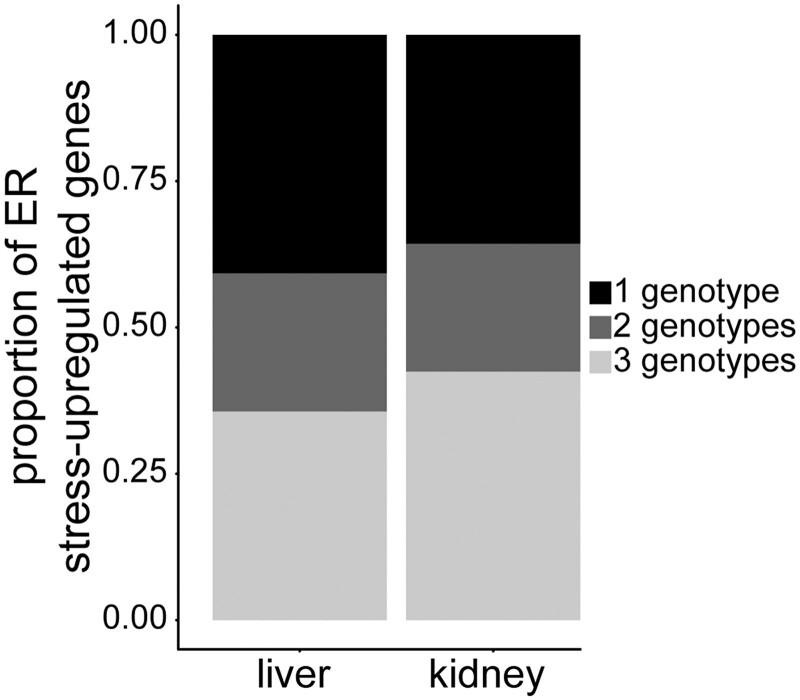
Genotype-specific expression post ER stress. Proportion of ER stress-upregulated genes that are shared in all 3 genotypes, shared in 2 genotypes, or unique to 1 genotype.

To determine how genetic background impacts variation in ER stress-induced gene expression in each tissue, we examined genotype-effects of ER stress on expression in liver and kidney. In either tissue, based on a 1.5-fold cutoff (FDR 5%), the majority of genes are upregulated post-ER stress in only 1 or 2 of the genotypes (Fig. 3). In liver, 2,330 (of 20,131, 11.5%) genes are significantly upregulated post-ER stress in at least one of the genotypes. Of these genes, 950 (41%) are uniquely upregulated in only 1 genotype, 550 (23%) are upregulated in 2 genotypes, and 830 (36%) are upregulated in all 3 genotypes (Fig. 3). In kidney, 2,718 (of 20,661, 13.2%) genes are significantly upregulated post-ER stress in at least one of the genotypes. Of these genes, 971 (36%) are uniquely upregulated in only 1 genotype, 594 (22%) are upregulated in 2 genotypes, and 1,153 (42%) are upregulated in all 3 genotypes (Fig. 3).

**Fig. 4.**
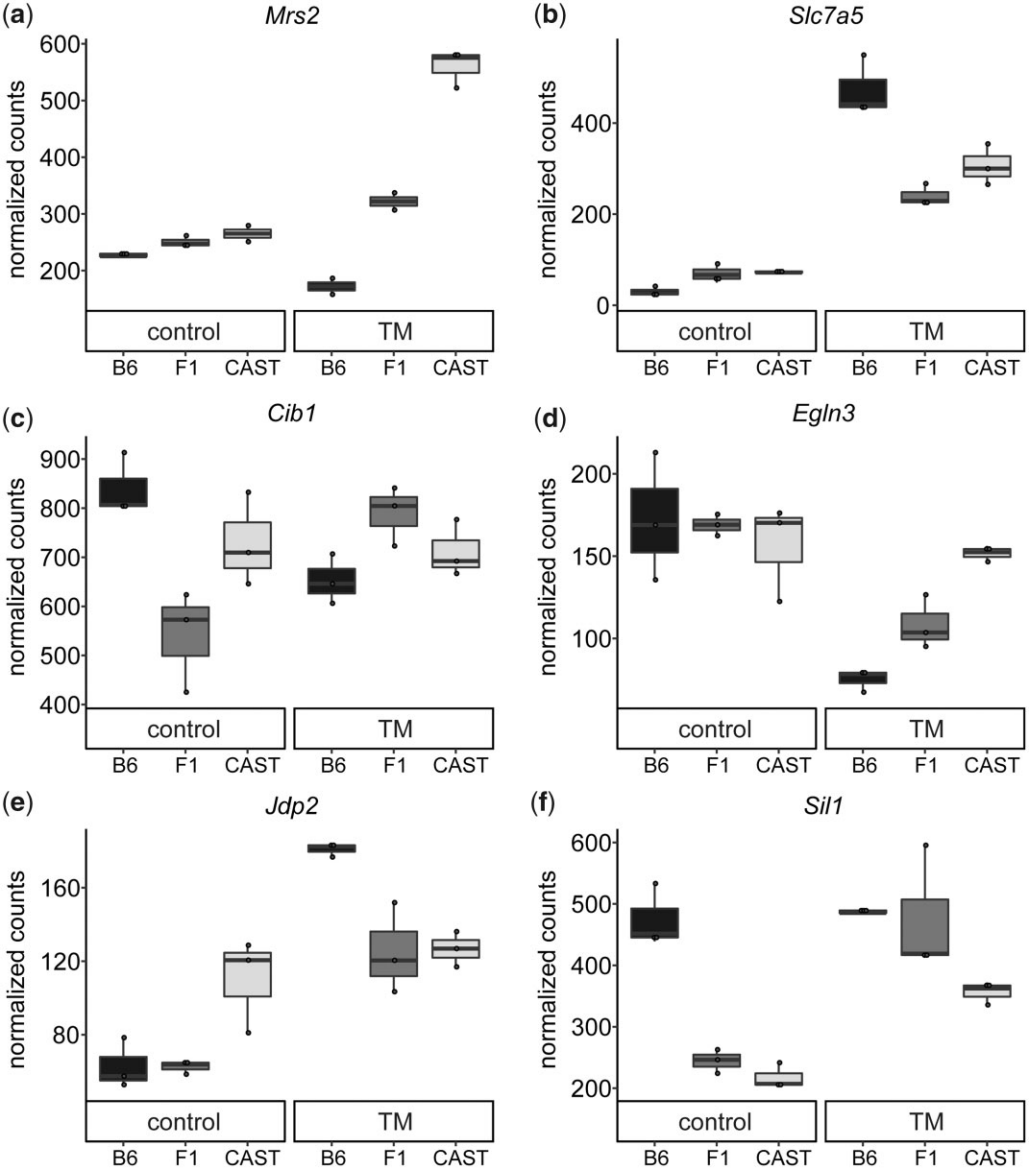
Variable expression of genes across genotypes post ER stress. Normalized counts for each genotype are plotted for control and TM conditions for a subset of genes that display a significant variable expression across the genotypes in (a) liver or (b–f) kidney. Each point represents a biological replicate. All plots show a significant genotype effect (*P*-adj < 0.05).

To identify genes with genotype-dependent ER stress-induced gene expression, we performed a likelihood-ratio test for each tissue. Ninety-one ER stress response genes in the liver were dependent on genetic background (91/5,143; 1.8% of ER stress-regulated genes) (*q* < 0.05) (Supplementary Table 8). There was no functional enrichment among these genotype-dependent ER stress response genes. Nevertheless, there were some striking observations. For example, many of these genes are involved in immunity, such as *Rsad2*, *Tlr12*, *Zc3hav1*, and *Nlrp6*. Other genes point to important genotype differences that may define how a genotype responds to stress. For example, *Mrs2*, which is involved in magnesium transportation into the mitochondria, shows one of the strongest genotype-dependent expressions post-ER stress (*P*-adj = 7.93 × 10^−06^) (Fig. 4a). Under control conditions, each genotype has similarly low levels of *Mrs2*. However, under ER stress, each genotype responds very differently (Log_2_FC: B6: -0.145; F1: 0.49; CAST: 1.23). *Mrs2* is required for the normal function of the mitochondrial respiratory complex, which has been linked to ER stress outcomes (Piskacek *et al.* 2009; Balsa *et al.* 2019; Knupp *et al.* 2019). *Mrs2* or any one of these immunity genes could play a role in the variable ER stress response given their genotype-dependent expression and their connection to the ER stress response (Todd *et al.* 2008; Bettigole and Glimcher 2015).

In kidney, 117 genes showed a genetic background-dependent response to ER stress (117/4,813; 2.4% of ER stress-regulated genes) (*q* < 0.05) (Supplementary Table 8). There was no functional enrichment among these genotype-dependent genes in the kidney. However, many of these genes have functions that can affect the ER stress response. Some of these processes include amino acid transport (*Slc7a5*), apoptosis (*Cib1, Egln3*), regulation of transcription (*Jdp2*), and nucleotide exchange factors (*Sil1*) (Fig. 4, b–f). Further functional validation will be required to confirm the impact these genes have on the variable ER stress response. Similar to liver, there are a number of genes that are involved in the immune response in kidney. However, the exact immunity genes with genotype-effects are unique to kidney, such as *Cdd36*, *Alcam*, and *Tapbpl*. For the 208 genes that display genotype-dependent expression in either liver or kidney, we asked which genotype had the highest or lowest expression of the 3 genotypes. We found F1 to be the least likely (23%, 95/416) to have the outlying expression level, indicating that in most genes that show a genotype-effect (77%, 321/416) there is a potential additive effect between the 3 genotypes where F1 expression is intermediate to B6 and CAST expression. There were a similar number of genes in both tissues that displayed genotype-dependent expression (liver: 91; kidney: 117). Between the 2 tissues, 9 genes showed a genotype effect in both tissues, which is greater than expected by random chance (*P* = 1.08 × 10^−05^). Of these 9 genes, some maintain similar expression patterns between the 2 tissues across the 3 genotypes, while some trend in different directions. For example, *Ces1f* displays genotype-dependent expression in both kidney and liver (kidney *q* = 0.034; liver: *q* = 7.18 × 10^−08^). However, the expression pattern is different between the 2 tissues. F1 *Ces1f* expression is the lowest of the 3 genotypes in liver, while F1 *Ces1f* expression is highest in kidney (Liver Log_2_FC: B6: −2.99, F1: −4.71, CAST: −3.22; Kidney: B6: −1.72, F1: −0.94, CAST: −2.32).

We next performed TF binding site enrichment analysis on genes variable across genetic background in liver and kidney (Supplementary Tables 8 and 4). For genes variable in liver, we found enrichment for TFs involved in diseases such as cancer (e.g. Evi1, *Z*-score = 9.9) and diabetes (e.g. Foxa2, *Z*-score = 8.5) (Wolfrum *et al.* 2004; Lugthart *et al.* 2008; Goyama and Kurokawa 2009). The most significant enrichment in kidney was for Nr3c1 (*Z*-score = 11.9). Nr3c1 binding sites are associated with 11 genes that show genotype-effect on ER stress response in kidney. Nr3c1 is involved in many processes, including inflammation, mRNA decay, and chromatin remodeling (Fryer and Archer 1998; Kadmiel and Cidlowski 2013; Cho *et al.* 2015). Similar to a previous study done in MEFs (Chow *et al.* 2015), there is also enrichment for genes involved in immunity and inflammation, like RELA and Nf-kappaB (Liver: *Z*-score = 4.9 and 10.7; Kidney: *Z*-score = 8.3 and 6.5).

### Identification of *cis-* and *trans-* regulatory variation

The variable ER stress transcriptional response could be due to *cis-* and *trans-* regulatory variation. These effects can impact a wide range of genes across different genetic backgrounds and tissue-types. A *cis-* regulatory variant influences the expression of a gene it is physically linked to. Due to this, allele-specific expression in a diploid, heterozygous animal (e.g. F1 hybrid mouse) is strong evidence for a *cis-* acting genetic variant in or near that expressed gene. An example of a *cis-* effect is a promoter polymorphism impacting the gene’s expression levels. A *trans-* regulatory variant influences an unlinked gene, often physically distant from the variant. As opposed to *cis-* acting variants, *trans-* acting variants affect both alleles equally, and consequently, differential expression of a gene between 2 inbred strains that cannot be explained by ASE in the F1 hybrid is most likely a result from a *trans-* acting variant. An example of a *trans-* effect is a polymorphism impacting a TF that can then alter the expression of a wide range of genes across the genome.

**Fig. 5.**
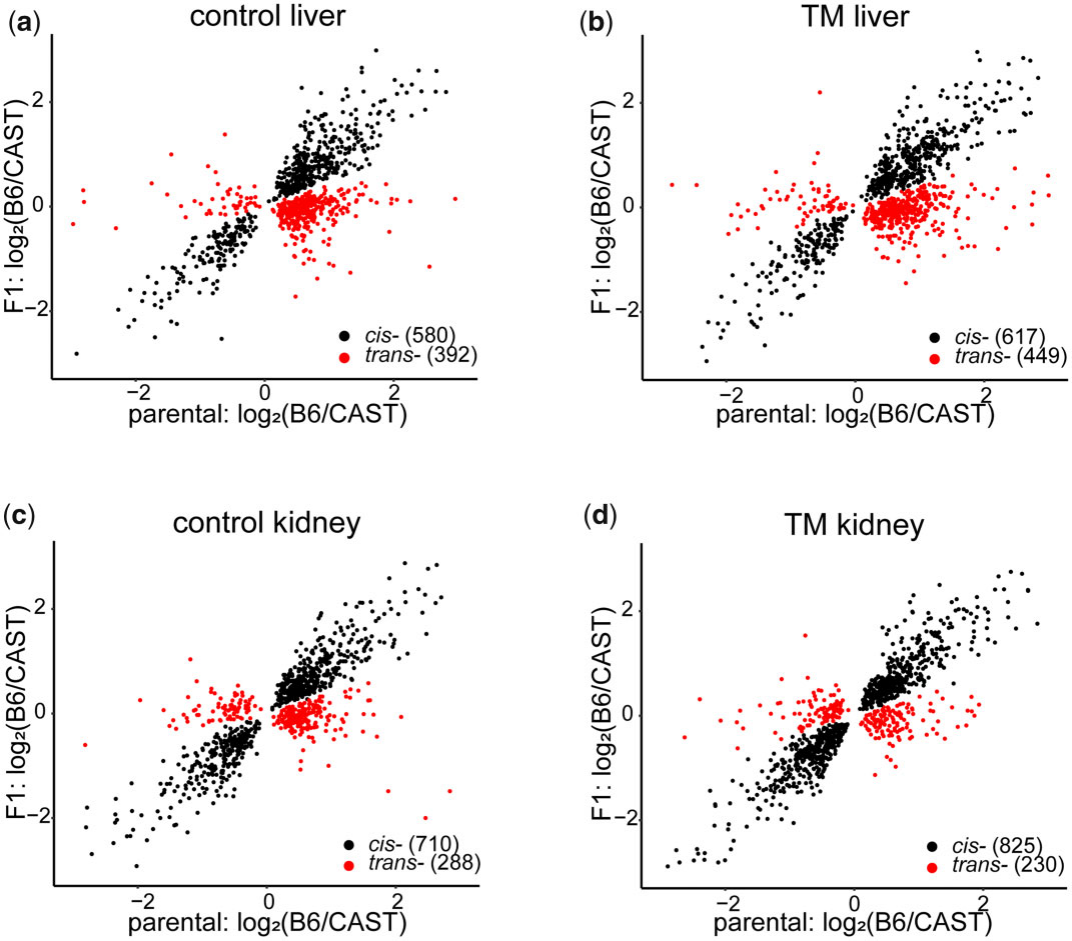
Expression ratios of genes with *cis-* or *trans-* effects. Log2(B6/CAST) plotted for either alleles in the F1 hybrid or parental expression in control liver (a), TM liver (b), control kidney (c) and TM kidney (d). Each point represents a gene displaying either a *cis-* or *trans-* regulatory effect. Numbers in parenthesis are the number of genes that display that particular regulatory effect.

The use of genetically divergent strains of mice and their hybrid F1 progeny allowed us to classify genetic effects as *cis-*, *trans-*, or a combination of the 2. Classification of *cis-* and *trans-* effects was performed using a previously published method (McManus *et al.* 2010; Chow *et al.* 2015). We performed *cis-* and *trans-* analyses on liver and kidney under control and TM conditions. Genes were assigned a *cis-* or *trans-* expression pattern (FDR = 0.1%; Supplementary Tables 9–11). In liver, under control conditions, 580 transcripts displayed a *cis-* effect and 392 transcripts displayed a *trans-* effect (Fig. 5a). Under TM conditions, 617 transcripts displayed a *cis-* effect and 449 transcripts displayed a *trans-* effect (Fig. 5b). In kidney, 710 transcripts displayed a *cis-* effect and 288 transcripts displayed a *trans-* effect under control conditions (Fig. 5c). Under TM conditions, 825 transcripts displayed a *cis-* effect and 230 transcripts displayed a *trans-* effect (Fig. 5d). The majority of effects we identified were classified as *cis-* regulatory variation.

In liver, there were many genes that displayed expression patterns matching both *cis-* and *trans-* effects. These effects can be acting in an additive manner (*cis-* + *trans-*) or in a nonadditive manner (*cis-* × *trans-*). Under control conditions, 304 transcripts displayed a *cis-* + *trans-* effect, while 124 transcripts displayed a *cis-* × *trans-* effect. Under TM conditions, 258 transcripts displayed a *cis-* + *trans-* effect, while 132 transcripts displayed a *cis-* × *trans-* effect (Supplementary Table 9). There were transcripts in kidney that also displayed a combination of *cis-* and *trans-* effects (Control: *cis-* + *trans-*: 229, *cis-* × *trans-*: 115; TM: *cis-* + *trans-*: 283, *cis-* × *trans-*: 121) (Supplementary Table 9). For the remaining analyses, we focused on genes that displayed only a *cis-* or *trans-* effect due to the difficulty of separating out the *cis-* and *trans-* effects of the *cis-* + *trans-* and the *cis-* × *trans-*categories. While these genes represent potentially interesting genes and patterns, they are not particularly informative in identifying patterns solely due to *cis-* or *trans-* effects.

### ER stress reveals cryptic regulatory variation unique to stress

**Fig. 6.**
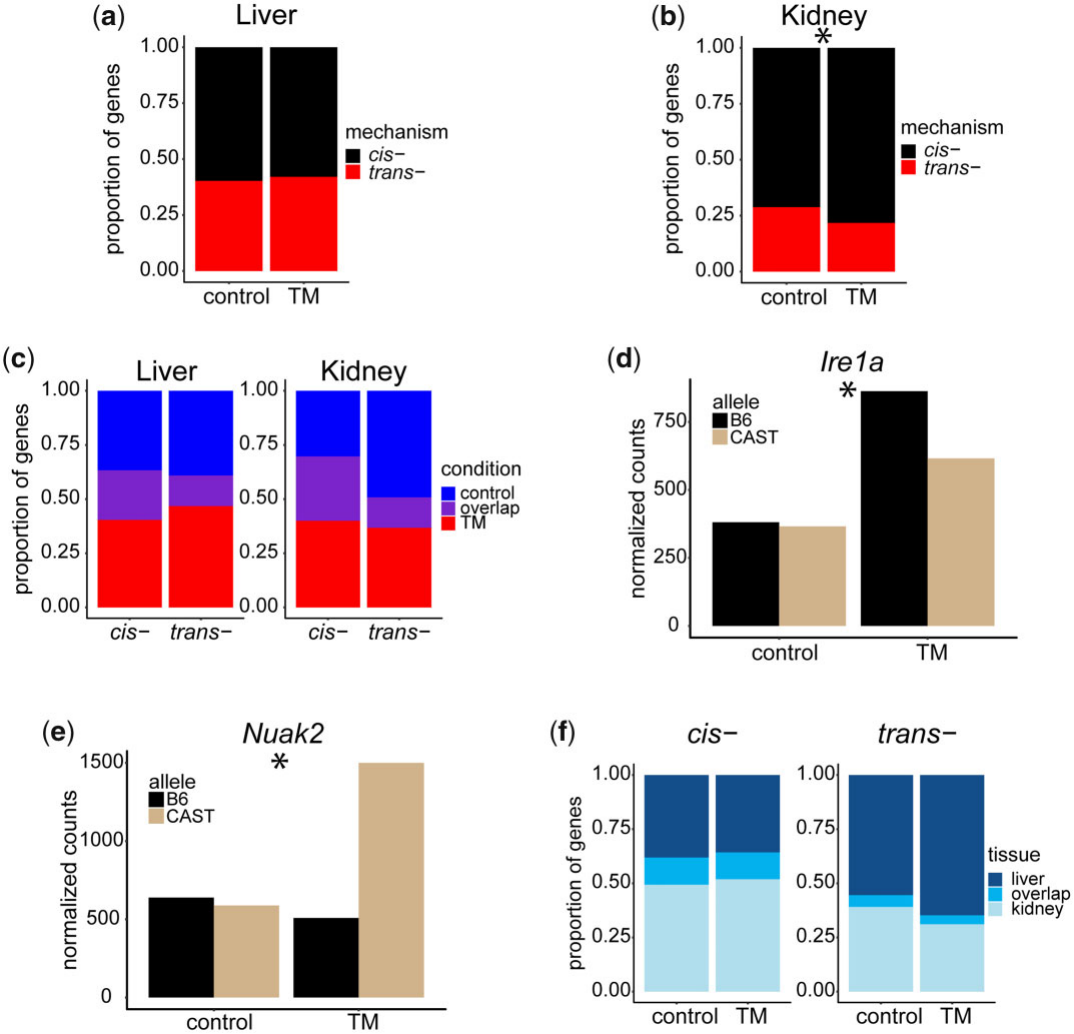
ER stress and tissue type reveal cryptic regulatory variation. Proportion of genes displaying a *cis-* or *trans-* effect in liver (a) and kidney (b). Proportion of genes with either a *cis-* or *trans-* effect in only stress conditions, only control conditions or both, for liver and kidney (c). Examples of genes displaying *cis-* regulatory effects only under stress conditions in F1 liver (d) and F1 kidney (e). Proportion of genes displaying a *cis-* or *trans-* effect seen in only liver, only kidney, or both (f). (b) *P < 0.00024. (d) *P = 0.0011. (e) *P < 0.00001.

To determine whether ER stress alters the contribution of *cis-* and *trans-* effects to regulatory variation, we compared the proportion of transcripts displaying a *cis-* or *trans-* effect in each tissue, under control and TM conditions. In liver, ER stress does not significantly alter the proportion of genes displaying a *cis-* effect (control: 0.59; TM: 0.58; *P* = 0.413) (Fig. 6a). However, in kidney, there is a small, but significant increase in the proportion of genes with a *cis-* effect under ER stress conditions (control: 0.71; TM: 0.78; *P* = 0.00024) (Fig. 6b).

Because the ER stress transcriptional response involves hundreds of transcripts, it is likely that the actual genes showing *cis-* and *trans-* patterns are different under stress. To address this, the overlap under control and TM conditions, in both the liver and kidney were analyzed for genes showing *cis-* and *trans-* regulatory variation. We observed both *cis-* and *trans-* regulatory variation that was unique to control or stress conditions or present under both conditions.

In liver, of the *cis-* effects detected under control or TM conditions, 37% (357/974) are unique to control, 40% (394/974) are unique to stress, and 23% (223/974) are common to both (Fig. 6c). Of the *trans-* effects detected under control or TM conditions, 39% (288/737) are unique to control, 47% (345/737) are unique to stress, and 14% (104/737) are common to both. The magnitude of the common *cis-* or *trans-* effects observed in both conditions were highly correlated (*r*^2^ = 0.86, *P* < 2.2 × 10^−16^) (Supplementary Fig. 7a), suggesting that this common, overlapping regulatory variation is not impacted by ER stress.

In kidney, 30% (359/1183) of *cis-* effects are unique to control, 40% (474/1183) are unique to stress, and 30% (351/1183) are common to both (Fig. 6c). Of the *trans-* effects detected in kidney, 49% (224/454) are unique to control, 37% (166/454) are unique to stress, and 14% (64/454) are common to both. The magnitude of the common *cis-* or *trans-* effects that were observed in both conditions were highly correlated (*r*^2^ = 0.91, *P* < 2.2 × 10^−16^) (Supplementary Fig. 7b) and likely not impacted by ER stress. The majority of genes that display a regulatory difference depend on the presence or absence of ER stress and those observed only in stress conditions may reveal critical components that might be responsible for the genotype-specific differences in the ER stress response.

In some cases, canonical UPR genes display regulatory variation only under stress conditions. For example, *Ire1α*, one of the main signal transducers of ER stress, displayed a strong *cis-* regulatory effect in the mouse liver that is only detectable under stress conditions (Fig. 6d) (*χ*^2^: *P* = 0.0011). Under control conditions, *Ire1α* is expressed at similar levels by the B6 and CAST allele. Once ER stress is induced, the B6 allele is expressed 1.5-fold higher than the CAST allele. There are 691 SNPs within a ± 2kb window of the *Ire1α* gene that differs between the B6 and CAST genotype. Any one or a combination of these SNPs could be contributing to this *cis-* regulatory difference. Genes that have not been implicated in the ER stress response, but show a strong, stress-specific transcriptional response, might represent novel UPR genes and pathways. For example, in the mouse kidney, the gene *Nuak2* displayed a strong *cis-* effect seen only under stress conditions (Fig. 6e) (*χ*^2^: *P* < 0.00001). While under control conditions the *Nuak2* alleles are equally expressed, but in the stressed F1 mouse kidney, the CAST allele is expressed 3-fold higher than the B6 allele.

### ER stress reveals cryptic regulatory variation unique to tissue type

We next asked whether there was tissue-specific regulatory variation. We investigated the overlap of genes that displayed *cis-* or *trans-* regulatory variation between liver and kidney in control and TM conditions. Of the genes that display a *cis-* effect under control conditions, 38% (436/1,146) are unique to liver, 49% (566/1,146) are unique to kidney, and 13% (144/1,146) are common to both (Fig. 6f). Of the genes that display a *cis-* effect under TM conditions, 36% (458/1,283) are unique to liver, 52% (666/1,283) are unique to kidney, and 12% (159/1,283) are common to both (Fig. 6f). The magnitude of the *cis-* effects observed in both tissues were moderately correlated (Control: *r*^2^ = 0.425, *P* < 2.2 × 10^−16^; TM: *r*^2^ = 0.408, *P* < 2.2 × 10^−16^) (Supplementary Fig. 8, a and b).

Of the genes that displayed a *trans-* regulatory effect under control conditions, 55% (357/644) are unique to liver, 39% (252/644) are unique to kidney, and 6% (35/644) are common to both (Fig. 6f). Of the genes that display a *trans-* regulatory effect under TM conditions, 65% (422/652) are unique to liver, 31% (203/652) are unique to kidney, and 4% (27/652) are common to both (Fig. 6f). The magnitude of the *trans-* effects observed in both tissues showed a small correlation only in control conditions (Control: *r*^2^ = 0.186, *P* = 0.009; TM: *r*^2^ = 0.126, *P* = 0.069) (Supplementary Fig. 8, c and d). Under control and TM conditions, we found that more genes with a *cis-* effect were common between the 2 tissues than genes with a *trans-* effect (Control: *χ*^2^*P* < 0.00001; TM: *χ*^2^*P* < 0.00001).

Under TM conditions, the majority of genes that displayed *cis-* or *trans-* regulatory variation were unique to either liver or kidney. Any one of these genes with a tissue- and stress-specific regulatory effect could be a gene involved in inter-individual variation in tissue-specific ER stress responses. For example, the genes *Lama5*, *Hnf4a*, *Scnn1b*, and *Pkd2* all display strong *cis-* regulatory variation under stress conditions in kidney that are not observed in the mouse liver. Each of these genes were previously implicated in kidney diseases, such as Liddle’s syndrome and Polycystic kidney disease (Wang *et al.* 2006; Cornec-Le Gall *et al.* 2017; Marable *et al.* 2018; Voskarides *et al.* 2018). The kidney is a tissue that relies heavily on protein transport and secretion. Proper ER function and response to ER stress plays a large part in kidney function. In fact, ER stress and aberrant protein trafficking is pathogenic in a large number of kidney diseases (Schaeffer *et al.* 2014). In liver, for genes such as *Sidt2* and *Adk*, they display *cis-* regulatory variation that is unique to the stressed liver and have been associated with human fatty liver disease (Bjursell *et al.* 2011; Gao *et al.* 2016). These genes with tissue-specific *cis-* regulatory variation are clear examples of how genetic variation has a differential impact across tissues.

### Tissue-specific effects on the regulatory variation of gene expression

**Fig. 7.**
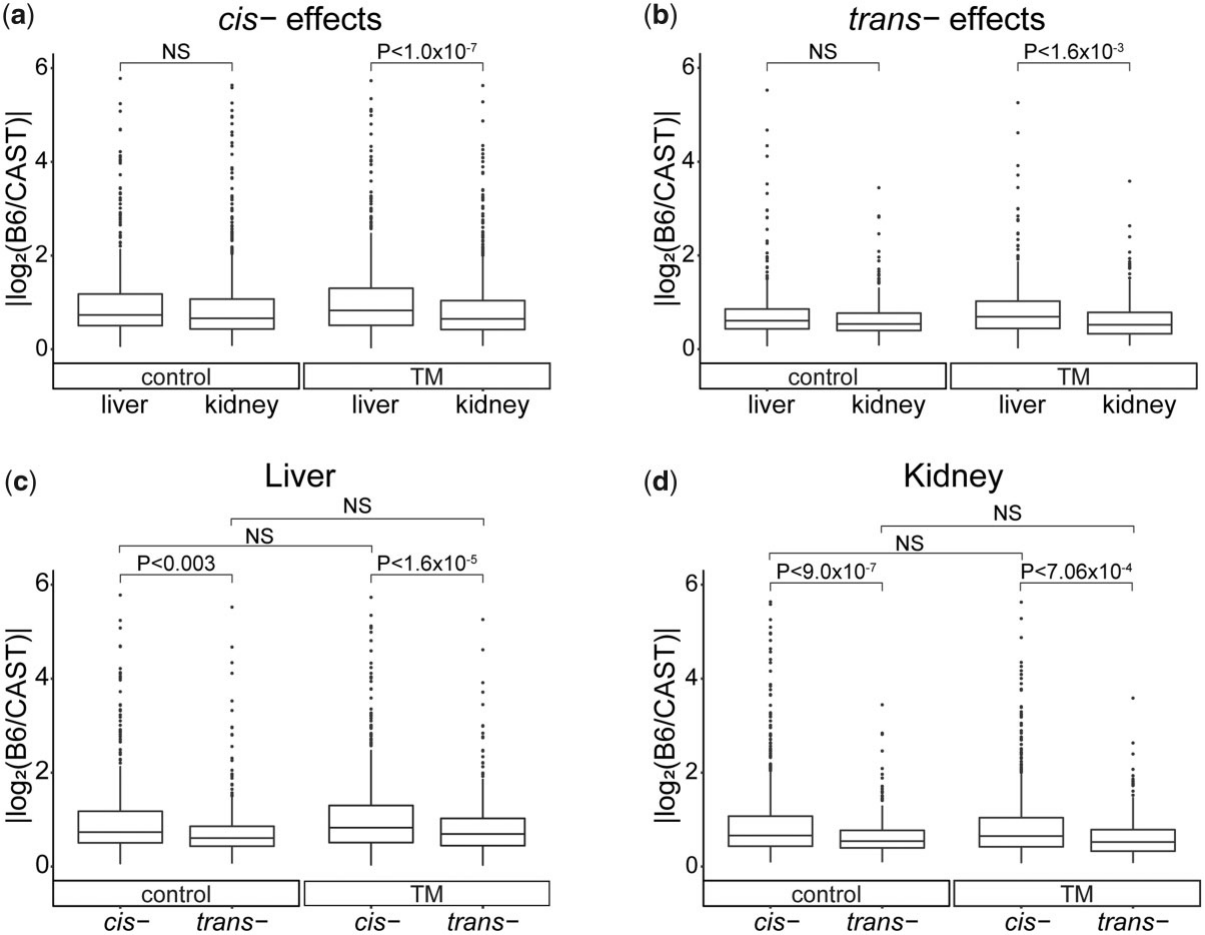
Impact of *cis-* and *trans-* effects on gene expression. Absolute log2 fold change of parental expression for genes displaying a regulatory effect. Comparing impact of *cis-* effects (a) or *trans-* effects (b) on gene expression in control or stress conditions. Comparing genes with a *cis-* or *trans-* effect in either control or stress conditions in liver (c) or kidney (d). Liver: control *cis-*: mean = 1.04, SD = 1.03 | control *trans-*: mean = 0.82, SD = 0.91 | TM *cis-*: mean = 1.14, SD = 1.09 | TM *trans-*: mean = 0.86, SD = 0.74; Kidney: control *cis-*: mean = 0.94, SD = 0.89 | control *trans-*: mean = 0.66, SD = 0.44 | TM *cis-*: mean = 0.87, SD = 0.74 | TM *trans-*: mean = 0.65, SD = 0.48. NS= not significant.

To determine the effect that tissue-type has on the magnitude of the effect of *cis-* and *trans-* regulatory variation on gene expression levels, we compared the ratio of the absolute fold change of the parental B6 expression to the parental CAST expression for each gene displaying *cis-* or *trans-* regulatory variation. We compared the effects of *cis-* and *trans-* regulatory variation between liver and kidney to better understand the impact of tissue type on the strength of a regulatory effect. Under control conditions, there is no difference between the strength of *cis-* effects between the 2 tissues (*P* = 0.170). However, under TM conditions, *cis-* effects in liver have a stronger effect on gene expression than in kidney (*P* < 1.0 × 10^−7^) (Fig. 7a). *trans-* effects, under control conditions, also showed no difference between tissues, but were stronger in liver than kidney under TM conditions (control: *P* = 0.20; TM: *P* < 1.6 × 10^−3^) (Fig. 7b). Within a condition, *cis-* effects on average are stronger than *trans-* effects, but tissue type and ER stress have different effects on the strength of regulatory effects. Genetic variation in the stressed liver has a stronger effect on transcription than in any other context.

In liver, under control and TM conditions, *cis-* regulatory differences have a stronger effect on gene expression levels than *trans-* regulatory differences (control: *P* < 0.003; TM: *P* < 1.6 × 10^−5^) (Fig. 7c). There was no difference in the magnitude of effect when comparing *cis-* or *trans-* regulatory variation across control and TM conditions in liver (*cis-: P* = 0.26; *trans-: P* = 0.93) (Fig. 7c). We found a similar pattern in kidney. *cis-* regulatory differences have a stronger effect on gene expression levels than *trans-* regulatory differences in control and TM conditions (control: *P* < 9.0 × 10^−7^; TM: *P* < 7.06 × 10^−4^) (Fig. 7d). Again, there was no difference in the effect when comparing *cis-* or *trans-* regulatory variation across conditions in kidney (*cis- P* = 0.25; *trans- P* = 0.98) (Fig. 7d).

### ER stress-induced change in allele-specific expression

**Fig. 8.**
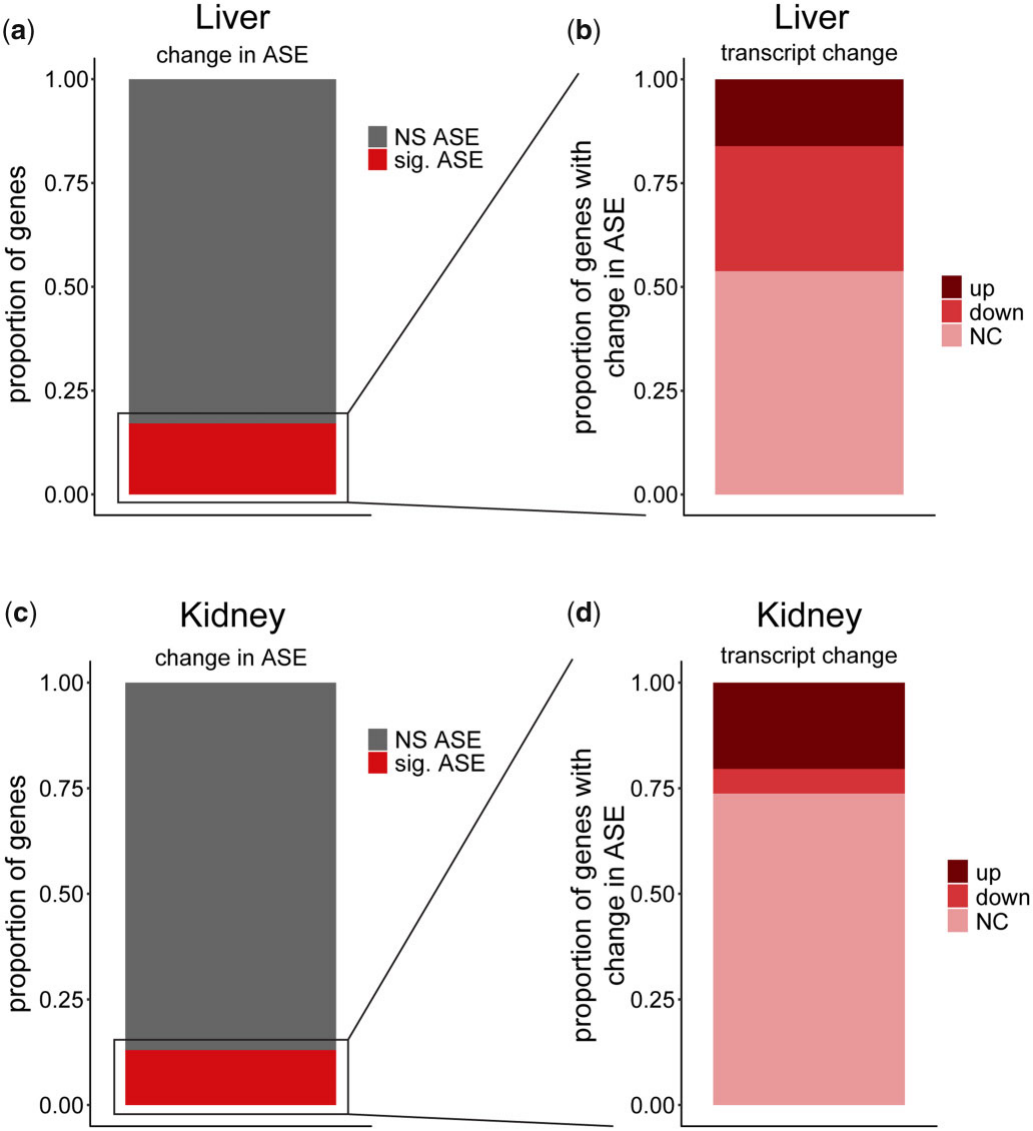
ASE and corresponding change in RNA transcript. Proportion of genes in the F1 displaying significant change in ASE in liver (a) and kidney (c). Proportion of genes with a significant change in ASE showing ER stress-induced increase in RNA transcripts, decrease, or no change in liver (b) and kidney (d).

Next, we tested whether ER stress changes the proportion of ASE—that is, do the 2 alleles respond differently to stress? To do this, we compared the allelic ratio in the F1 under stress and control conditions (Fisher’s exact test; 5% FDR). Overall, the ASE patterns were normally distributed and most genes showed equal allelic expression (Supplementary Fig. 9). A significant change in ASE post-ER stress was observed in 17% and 13% of expressed transcripts in liver (970/5669), and kidney (1010/7764), respectively (Fig. 8, a and c) (Supplementary Table 12). The liver displayed a higher proportion of genes with a change in ASE under stress (*χ*^2^; *P* = 0.00001). Change in ASE in both tissues was driven equally by CAST and B6 alleles, indicating that there is no unexpected hybrid effect (Supplementary Fig. 1). In liver, of the 970 genes that displayed a significant change in ASE, 191 (/970, 20%) genes displayed a *cis-* effect under control conditions and 243 (/970, 25%) genes under TM conditions (Supplementary Fig. 10). Only 113 (/970, 12%) genes displayed a *trans-* effect in control conditions and only 104 (/970, 11%) genes displayed a *trans-* effect in TM conditions (Supplementary Fig. 10). In kidney, of the 1,010 genes that displayed a significant change in ASE, 185 (/1010, 18%) displayed a *cis-* effect under control conditions and 227 (/1010, 22%) under TM conditions. Only 63 (/1010, 6%) genes displayed a *trans-* effect in control conditions and only 51 (/1010, 5%) genes displayed a *trans-* effect in TM conditions (Supplementary Fig. 10). Significant ASE genes classified as a combination of *cis-* and *trans-* were not discussed in this paper.

**Fig. 9.**
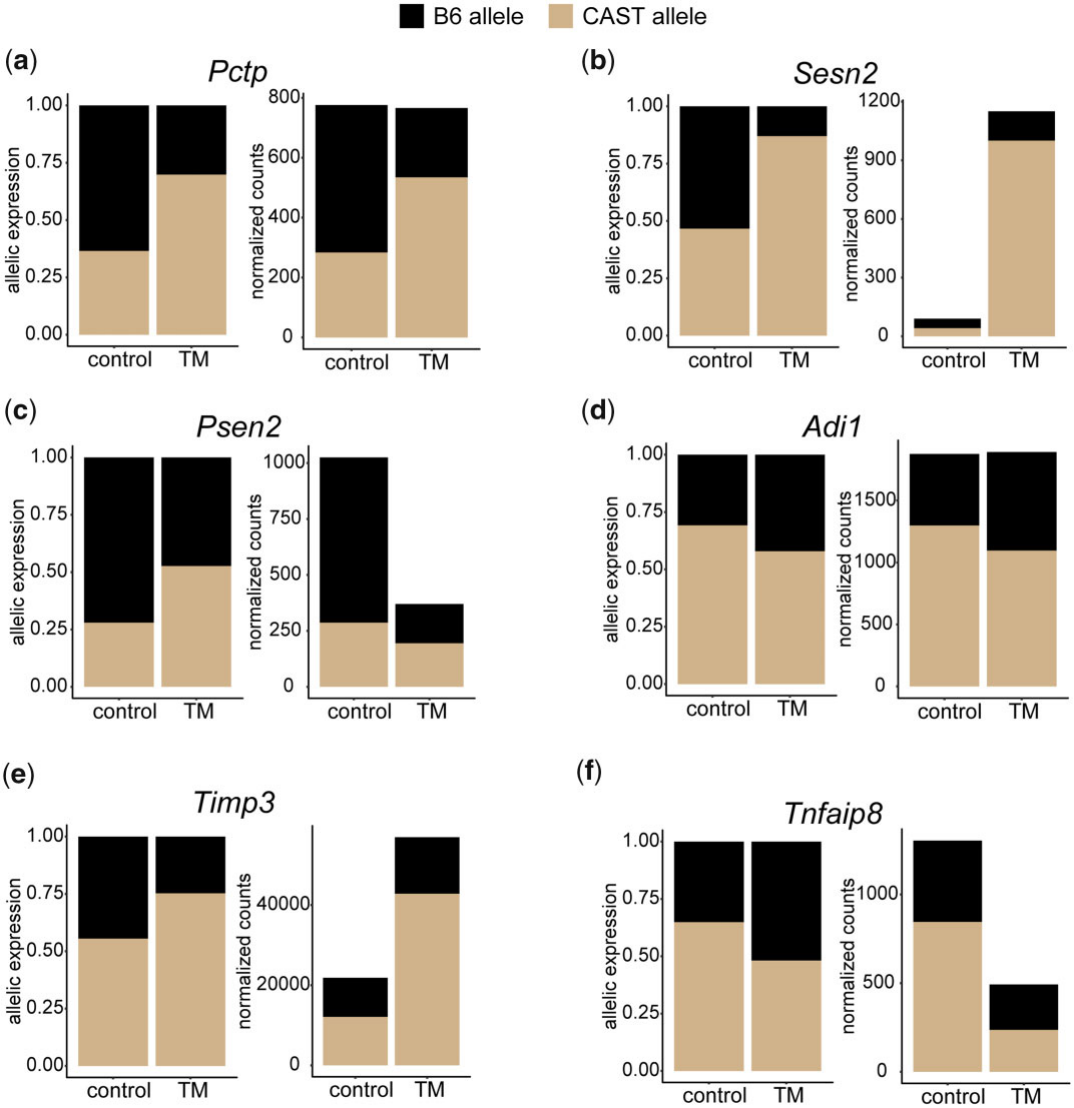
Change in ASE and transcript levels across stress conditions. ASE and total RNA expression levels are plotted. Genes that display ER stress-induced change in ASE in liver (a–c) or kidney (d–f). (a) and (d) show no change in total transcript levels, (b) and (e) show an increase in total transcript level, and (c) and (f) show a decrease in total transcript levels.

Genes that exhibit a change in ASE and transcript level post ER stress fall in both up- and downregulated categories (Liver: 35% upregulated 156/448, 65% downregulated 292/448; Kidney: 78% upregulated 206/265, 22% downregulated 59/265) (Fig. 8, b and d). In all cases, the B6 and CAST alleles contributed equally to changes in ASE (Supplementary Fig. 11, a and b). However, the majority of genes that show an ER stress-induced change in ASE do not exhibit a change in their total transcript abundance (liver: 522/970 or 54%; kidney: 745/1010 or 74%) (Fig. 8, b and d). This pattern was observed in a previous study performed in fibroblasts (Chow *et al.* 2015). For example, under control conditions, the B6 allele of the gene Phosphatidylcholine transfer protein (*Pctp*) accounts for 63% of allelic expression, while under TM conditions, the B6 allele accounts for only 30% of allelic expression (Fisher’s exact; *q* = 0.00001) (Fig. 9a). While the ratio of expression between alleles is significantly changed under TM conditions, the net result is no significant change in total RNA transcript levels, suggesting a possible compensatory mechanism between the 2 alleles.

Genes that show a significant change in ASE and in transcript levels post-ER stress are of particular interest, as this suggests differential allelic response to stress. *Sesn2*, which is involved in a variety of different stress responses (Lee *et al.* 2010), showed one of the most significant changes in ASE (Fisher’s exact; *q* < 0.00001). Under control conditions, the B6 and CAST allele in the F1 hybrid mouse are expressed at equal levels (B6: 0.53; CAST: 0.47). Total *Sesn2* transcript responded to TM conditions with a 13-fold increase. At the allelic level, the CAST allele is increased 24-fold, while the B6 allele is only increased 3-fold (TM: B6: 0.13, CAST: 0.87) (Fig. 9b). The large increase of the CAST allele is driving the *Sesn2* transcriptional response to TM-induced ER stress in the F1. In the parental strains, there is a similar bias in terms of *Sesn2* upregulation post-ER stress. The B6 parental strain has a 6-fold increase in *Sesn2* expression while the CAST parental strain has a 22-fold increase. This strong parental and allelic response indicates that *Sesn2* contains a strain- and ER stress-specific *cis-* element that drives differential transcriptional response to ER stress and potentially other stress stimuli such as hypoxia and reactive oxygen species (Lee *et al.* 2010). We see similar patterns for genes that are downregulated post-ER stress. *Presenilin 2* (*Psen2*), which cleaves proteins such as APP (amyloid-beta precursor protein) (De Strooper *et al.* 2012) and has been shown to cause Alzheimer’s disease, displays a change in ASE (Fisher’s exact; *q* = 0.00001) and a 2.7-fold decrease in RNA transcript levels (Fig. 9c). Under control conditions, the B6 allele is more highly expressed (B6: 0.72; CAST: 0.28). Under stress conditions, the *Psen2* B6 allele decreases 4.2-fold in expression while the CAST allele only decreases 1.6-fold. The greater reduction of the B6 allele results in near equal expression levels of the 2 alleles under stress conditions (B6: 0.47; CAST: 0.53). The mirrored in the parental strains as B6 has a 2.2-fold decrease in *Psen2* expression while CAST has only a 1.1-fold decrease. This again indicates a strain- and ER stress-specific genetic element that drives this differential response of *Psen2* to ER stress in different genetic backgrounds. Similar patterns are observed in kidney with a wide range of genes (Fig. 9, d–f).

**Fig. 10.**
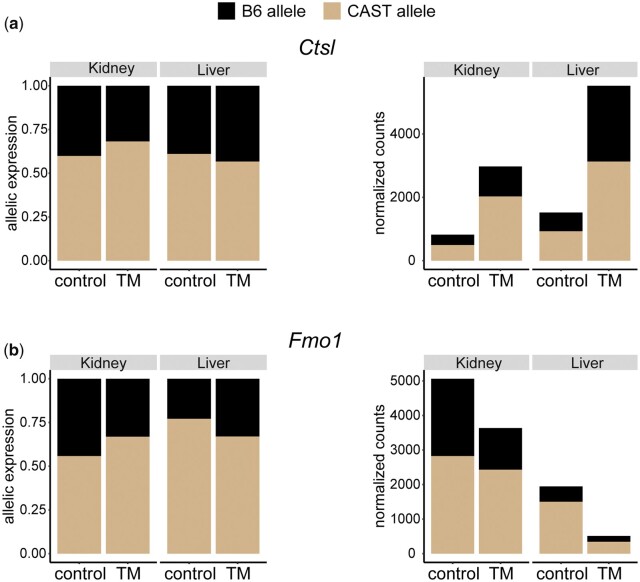
Variable magnitude in ASE across tissues. Examples of genes that show ASE in both tissues, but differ in the magnitude of that ASE in a tissue-dependent manner. An example of an upregulated gene (a) and a downregulated gene (b). ASE and total RNA expression levels are plotted.

The majority of ASE post ER stress is tissue specific. Only 210 transcripts display a change in ASE post-ER stress common to both tissues (Liver: 210/970, 22%; Kidney: 210/1010, 21%) (Supplementary Fig. 12). For these common genes, tissue type has a strong effect on the magnitude of the ASE changes post ER stress, in line with what we observed with tissue-specific changes in magnitude of *cis-* and *trans-* regulatory variation. For example, in *Cathepsin L* (*Ctsl*) (Fig. 10a), the CAST allele is more ER stress responsive in kidney, while in liver, the B6 allele is more responsive. *Ctsl*, which is involved in lysosomal protein degradation, is upregulated in both liver (FC = 4.3) and kidney (FC = 3.2) post-ER stress. *Ctsl* also shows a change in ASE in liver (*q* = 0.017) and kidney (*q* = 0.0002). However, in liver, the CAST allele was responsible for only 55% of the increase in expression levels, but in kidney, the CAST allele was responsible for 71% of the increase in expression levels. This pattern was also observed in downregulated genes. *Flavin containing dimethylaniline monoxygenase 1* (*Fmo1*) (Fig. 10b), which is involved in the oxidation reduction process, is downregulated in both liver (FC = −3.54) and kidney (FC = −1.5) and shows a change in ASE in liver (*q* = 7 × 10^−5^) and kidney (*q* < 0.00001). The CAST allele in liver accounts for 80% of the expression downregulation, but only 28% of the downregulation in kidney.

## Discussion

The ER stress and large UPR transcriptional response provides a unique opportunity to study how G×E interactions can alter gene expression levels across different tissues. We took advantage of 2 genetically diverse mouse strains, B6 and CAST, and their F1, and induced a strong in vivo ER stress transcriptional response. This provided the opportunity to study how stress and tissue type impacts the effect of genetic variation. We uncovered genes that showed variable transcript levels in a genotype × tissue × stress manner. These genes implicated networks and pathways that could contribute to the variable ER stress response. In addition, the F1 hybrid gave us the ability to uncover the *cis-* and *trans-* regulatory variation that is impacted by stress and tissue type. We discovered that most *cis-* and *trans-* regulatory variation is context specific, with most unique to only 1 context. Altogether our results provide a better understanding for how genetic background and tissue type impacts the genetic architecture of the inter-individual transcriptional response to ER stress in mouse and how different genotypes respond to different environments.

We previously used MEFs to assay how a complex genetic architecture influences the transcriptional response to ER stress across different genetic backgrounds (Chow *et al.* 2015). Here, we utilized an in vivo mouse model to identify how these patterns change when comparing across different tissues. In the MEF study, upregulated genes most significantly influenced by genetic background were enriched for roles in inflammation (Chow *et al.* 2015). However, in this current study, we find no enrichment for any particular function in the genotype-dependent genes, in either tissue. In contrast to the MEF study, these genes are involved in such a wide array of functions, that there is no enrichment. Much of the variability in the ER stress response likely stems from these disparate genes. However, we did find commonalities in these genes, such as many being involved in immunity displaying a genotype-effect. In addition, we find genes with roles clearly linked to the ER stress response such as apoptosis, protein transport, regulation of transcription, and amino acid transport. This demonstrates the strong impact that genotype has on how different genes respond to ER stress in different tissues. In addition, this highlights the strength of an in vivo study which utilizes different tissues to uncover greater depth to the variable ER stress response.

This study emphasizes the strong effect that tissue type has on how genetic variation impacts the transcriptional response to ER stress. These differences are observed in the number of regulatory effects and their impact on transcript levels across tissues. A greater number of genes displayed variation across tissue-type post-ER stress than genes that displayed variation across genotypes post-ER stress. Even between the highly divergent strains of B6 and CAST, tissue identify has a stronger effect than genotype. In addition to tissue differences, this work identifies commonalities across tissues and genetic backgrounds and can provide insight into what is integral to the ER stress response. For example, we found genes involved in ribosome biogenesis and the nucleolus upregulated in response to ER stress independent of tissue-type and genetic background. Increasingly, there is evidence suggesting a role for ribosome biogenesis and the nucleolus in the ER stress response (Yang *et al.* 2018; Chen and Stark 2019; Pecoraro *et al.* 2020). Further functional studies with the addition of more tissues and genetic backgrounds can better elucidate the role and function of the nucleolar stress response in the context of ER stress.

The genotype- and tissue- dependent genes highlighted in this study likely represent a small subset of the genes that make up the complete story of the variable ER stress response. The use of arbitrary cutoffs results in the exclusion of true effects at subthreshold levels. Despite this, we are confident that the genes we are identifying in this study with the most significant levels have the strongest impacts on gene expression and the variable ER stress response. Inclusion of more genetic backgrounds and tissue-types will be necessary to build a more complete picture of the genes underlying the genotype-dependent ER stress response.

The ER stress response is implicated in many different diseases such as Alzheimer’s disease, type II diabetes, ALS, atherosclerosis, and cancer (Oyadomari *et al.* 2002; Song *et al.* 2008; Auf *et al.* 2010; Wang, Popko *et al.* 2011; Ozcan and Tabas 2012; Wang *et al.* 2014 ). Each of these diseases are unique in their tissue of origin. In addition, each individual diagnosed with an ER stress-related disease will have different genetic backgrounds. The tissue-dependent ER stress response observed in this study illustrates how future studies involving ER stress and disease should investigate the disease-relevant tissue in the context of different genetic backgrounds, potentially uncovering novel tissue-specific effects and mechanisms. For example, the combination of tissue-type and genetic background revealed Nuak2 to have differential allelic regulation under ER stress conditions (Fig. 6e). Nuak2 belongs to the AMPK protein kinase family and has mainly been linked to cancer (Sun *et al.* 2013; Yuan *et al.* 2018). Understanding Nuak2 and its role in AMPK regulation and ER stress can provide insight into the role of Nuak2 in human disease such as cancer.

This study utilized genetic variation present in different strains of mice to demonstrate the strong impact that genetic background and tissue type have on cellular processes such as the ER stress response. We detected numerous ER stress- and tissue-specific responses in expression levels, regulatory variation, and allele-specific effects. The majority of these findings would have been missed if only studying 1 genotype, condition, or tissue. Future studies can reveal even more complex interactions that affect transcriptional levels by incorporating more variables, such as cell type, additional tissues, and other cellular stressors. This type of analysis provides better predictive power for the dynamic effects that genetic variation has on transcriptional levels in different tissues and contexts.

## Data availability

The data underlying this article are available in the sequence read archive (SRA), and can be accessed with BioProject ID PRJNA715325. Data were also uploaded to gene expression omnibus (GEO) and can be accessed with GSE189419. Code used for analysis can be found at Zenodo: https://doi.org/10.5281/zenodo.6458482 (accessed 2022 May 3).

Supplemental material is available at *G3* online.

## Supplementary Material

jkac104_Supplementary_Data

jkac104_Supplementary_Figure_S1

jkac104_Supplementary_Figure_S2

jkac104_Supplementary_Figure_S3

jkac104_Supplementary_Figure_S4

jkac104_Supplementary_Figure_S5

jkac104_Supplementary_Figure_S6

jkac104_Supplementary_Figure_S7

jkac104_Supplementary_Figure_S8

jkac104_Supplementary_Figure_S9

jkac104_Supplementary_Figure_S10

jkac104_Supplementary_Figure_S11

jkac104_Supplementary_Figure_S12

jkac104_Supplementary_Table_S1

jkac104_Supplementary_Table_S2

jkac104_Supplementary_Table_S3

jkac104_Supplementary_Table_S4

jkac104_Supplementary_Table_S5

jkac104_Supplementary_Table_S6

jkac104_Supplementary_Table_S7

jkac104_Supplementary_Table_S8

jkac104_Supplementary_Table_S9

jkac104_Supplementary_Table_S10

jkac104_Supplementary_Table_S11

jkac104_Supplementary_Table_S12
