# Supplementary material for: The dynamic effect of genetic variation on the in vivo ER stress transcriptional response in different tissues
Source: G3 (Bethesda). 2022 Apr 29;12(6):jkac104. doi: 10.1093/g3journal/jkac104 (PMC9157157; doi:10.1093/g3journal/jkac104)
Supplement: jkac104_Supplementary_Data [file jkac104_supplementary_data.docx]

**Supporting information**

**Supplementary Figure S1. No genotype bias in genes with significant change in ASE.** The proportion of genes with an ER stress-induced change in ASE that show an increase of the B6 or CAST allele in liver (A) or kidney (B).

**Supplementary Figure S2. MA plots of tissue difference in expression for each genotype.** MA plots generated from the tissue-dependent comparison performed in Deseq2. MA plots were generated for the tissue comparison in B6 (A), CAST (B), and F1 (C). Blue colored points are genes with adjusted p-values less than 0.1.

**Supplementary Figure S3. Classification of *cis-* and *trans-* regulatory effects.** Statistical tests, cutoffs, and classifications used to assign a gene a regulatory effect.

**Supplementary Figure S4. Confirmation of ER stress induction.** *Xbp1* transcript is spliced by IRE1 when misfolded proteins are present in the ER. Spliced *Xbp1* transcript is a marker of ER stress. RT-PCR for spliced and unspliced *Xbp1* transcript in both control and TM mice for both liver and kidney (A). Upper band represents unspliced *Xbp1* at 183 bp and lower band represents spliced *Xbp1* at 157 bp. *Bip* is upregulated under ER stress conditions and is another hallmark of the UPR. *BiP* levels were measured by qRT-PCR in liver and kidney from control and TM injected mice (B).

**Supplementary Figure S5. Correlation of post ER stress expression between tissues.** Correlation of Log2(TM/control) of the genes expressed in liver and kidney in B6 (A), CAST (B), and F1 (C). Blue line is regression line between the two tissues. Red dots represent genes with a significantly different expression pattern between the two tissues.

**Supplementary Figure S6. Overlap of genes with or without a tissue-effect between the three genotypes.** Venn Diagram between the three different genotypes for genes that displayed a tissue-effect (A) and for genes that displayed a tissue-independent effect (B).

**Supplementary Figure S7. Genes that show the same *cis-* or *trans-* regulatory effect under both conditions are strongly correlated in their magnitude.**  Data for liver (A) and kidney (B). ER stress does not affect the regulation of these genes (Liver: *cis-*: 223, *trans-*: 104; Kidney: *cis-*: 351, *trans-*: 64).

**Supplementary Figure S8. Genes that show the same *cis-* or *trans-* regulatory effect in both tissues are slightly correlated in their magnitude.** *cis-* effects observed in tissues under control (A) and TM (B) conditions. *trans-* effects in both tissues under control (C) and TM (D) conditions.

**Supplementary Figure S9. ASE distribution shows equal allelic expression patterns.** Distribution of ASE ratios for each tissue and condition. The proportion of total expression due to the B6 allele, as opposed to the CAST allele, is plotted for control liver (A), TM liver (B), control kidney (C), and TM kidney (D).

**Supplementary Figure S10. Regulatory classifications of genes with significant ASE.** Of all the genes that display a significant change in ASE, we categorized them based on if they showed a *cis-* regulatory effect only, *trans-* regulatory effect only, or other in either control or TM conditions for liver (A) and kidney (B).

**Supplementary Figure S11. No genotype bias in genes with significant change in ASE.** The proportion of genes with an ER stress-induced change in ASE that show an increase of the B6 or CAST allele in liver (A) or kidney (B). No genotype bias in genes that display significant change in ASE and significant change in RNA transcript levels in liver (C) or kidney (D). For genes downregulated in kidney, there is a trend towards the CAST allele contributing more towards these ASE effects. However, due to the low number of genes in this category, this bias is not significant (p=0.136).

**Supplementary Figure S12. The majority of genes showing ER stress-induced ASE are tissue-specific.** The proportion of genes that display a significant change in ASE post-ER stress that in liver, kidney, or both.

**Supplementary Table S1. ER stress induced upregulated genes.**

**Supplementary Table S2. ER stress induced downregulated genes.**

**Supplementary Table S3. GO enrichment analysis of ER-stress induced genes.**

**Supplementary Table S4. Transcription factor binding site enrichment analysis.**

**Supplementary Table S5. Tissue effects on ER stress induced expression.**

**Supplementary Table S6. GO enrichment of tissue-effect genes.**

**Supplementary Table S7. GO enrichment of genes unaffected by tissue.**

**Supplementary Table S8. ER stress-induced genes with genotype effect.**

**Supplementary Table S9. Counts for each regulatory mechanism.**

**Supplementary Table S10. B6xCAST F1 liver.**

**Supplementary Table S11. B6xCAST F1 kidney.**

**Supplementary Table S12. Allele specific expression.**
