# Supplementary figures and images for: The dynamic effect of genetic variation on the in vivo ER stress transcriptional response in different tissues
Source: G3 (Bethesda). 2022 Apr 29;12(6):jkac104. doi: 10.1093/g3journal/jkac104 (PMC9157157; doi:10.1093/g3journal/jkac104)

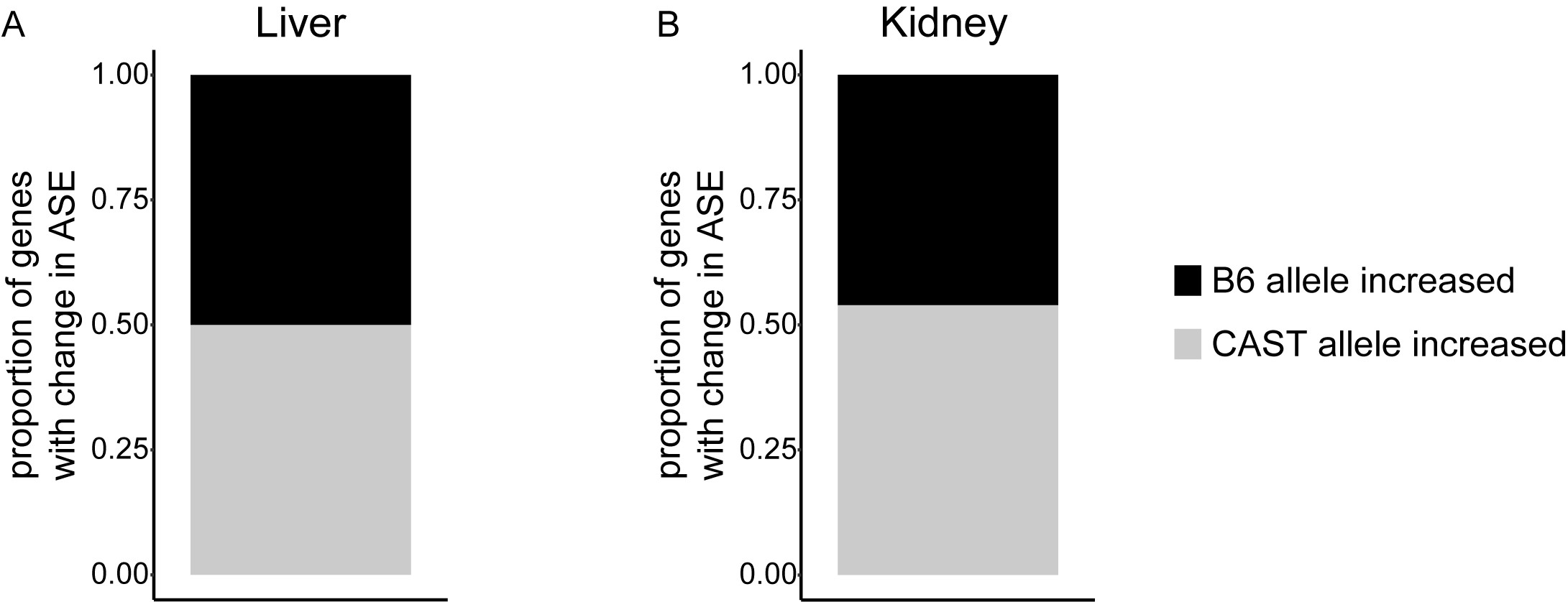

Supplement: jkac104_Supplementary_Figure_S1 [file jkac104_supplementary_figure_s1.jpeg]

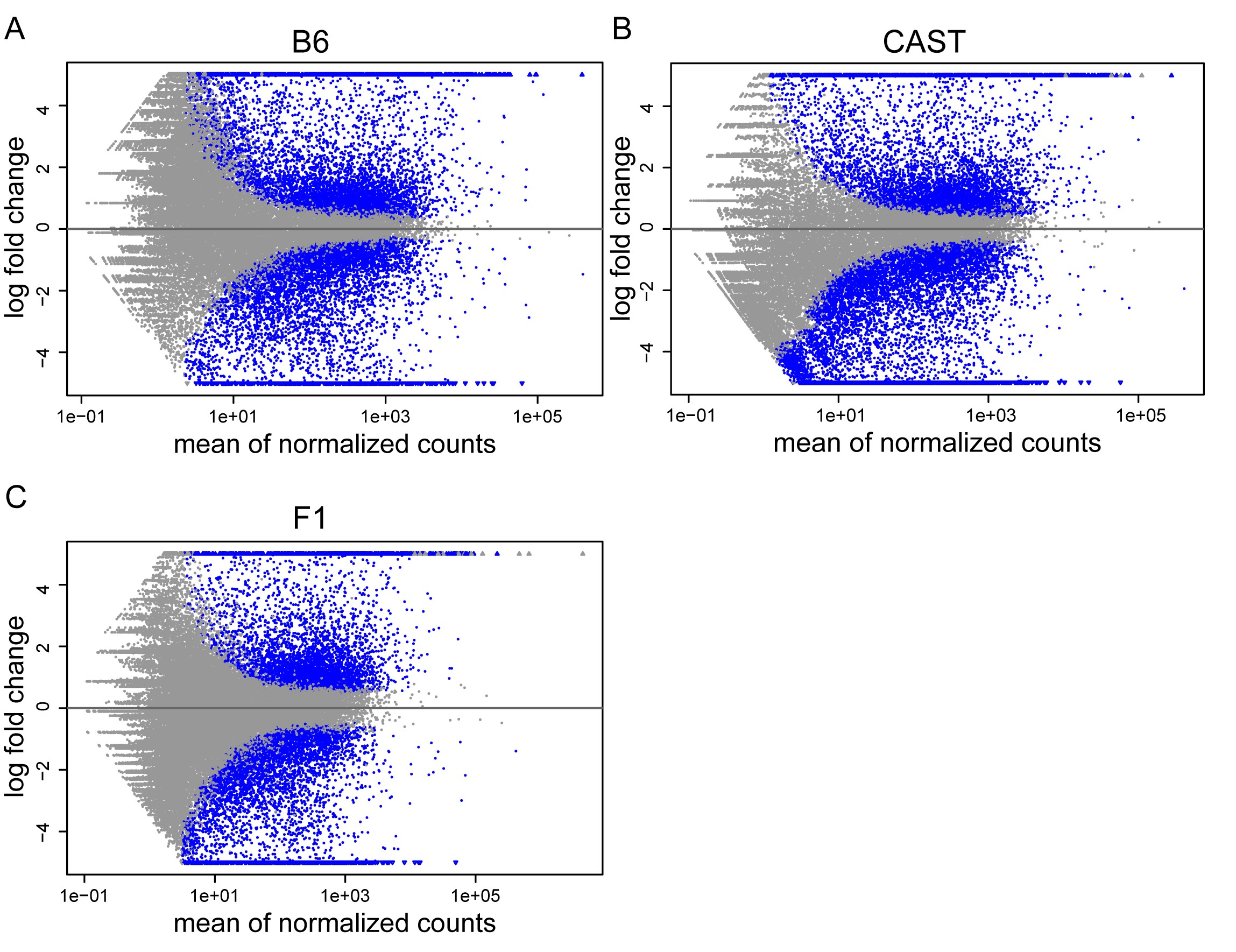

Supplement: jkac104_Supplementary_Figure_S2 [file jkac104_supplementary_figure_s2.jpeg]

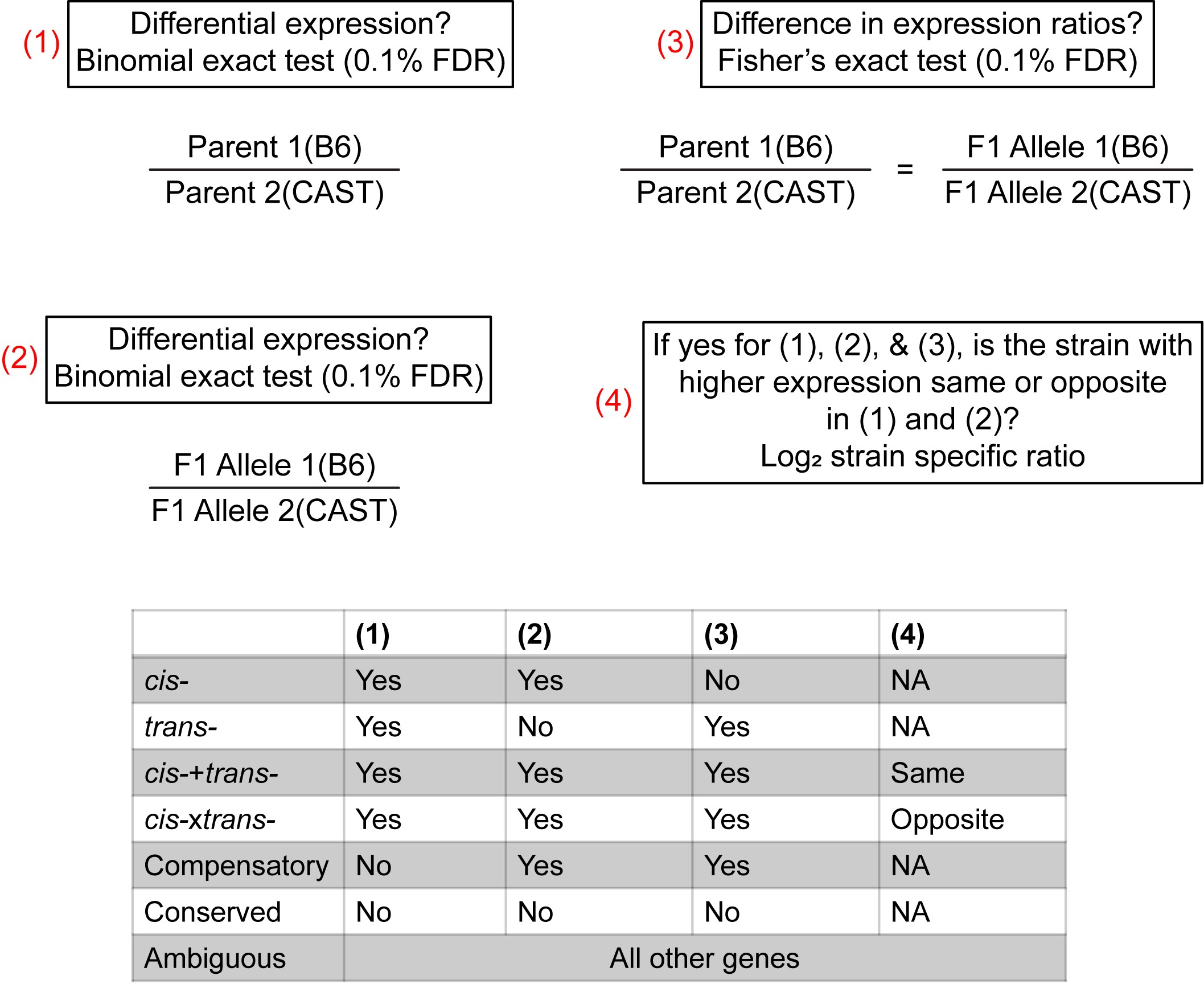

Supplement: jkac104_Supplementary_Figure_S3 [file jkac104_supplementary_figure_s3.jpeg]

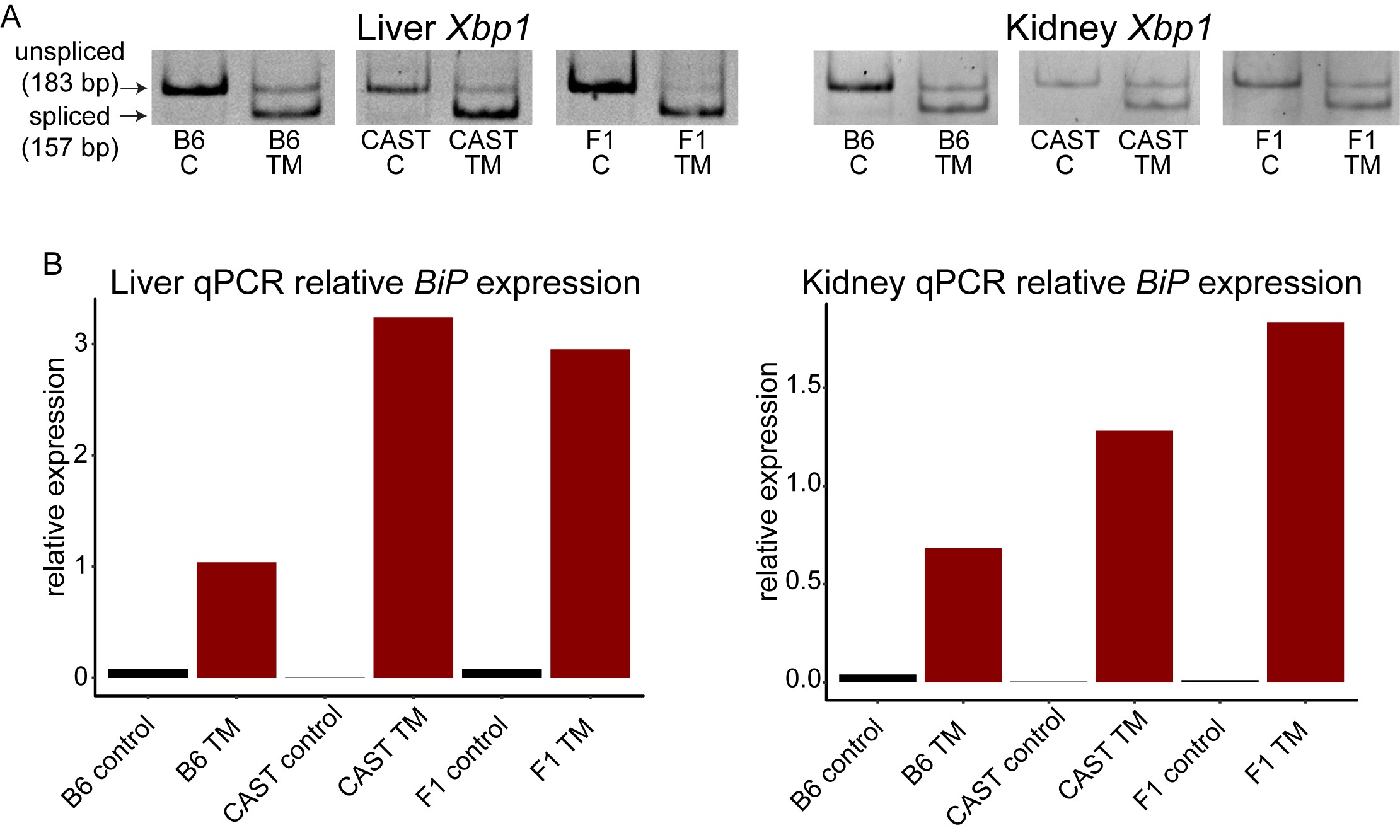

Supplement: jkac104_Supplementary_Figure_S4 [file jkac104_supplementary_figure_s4.jpeg]

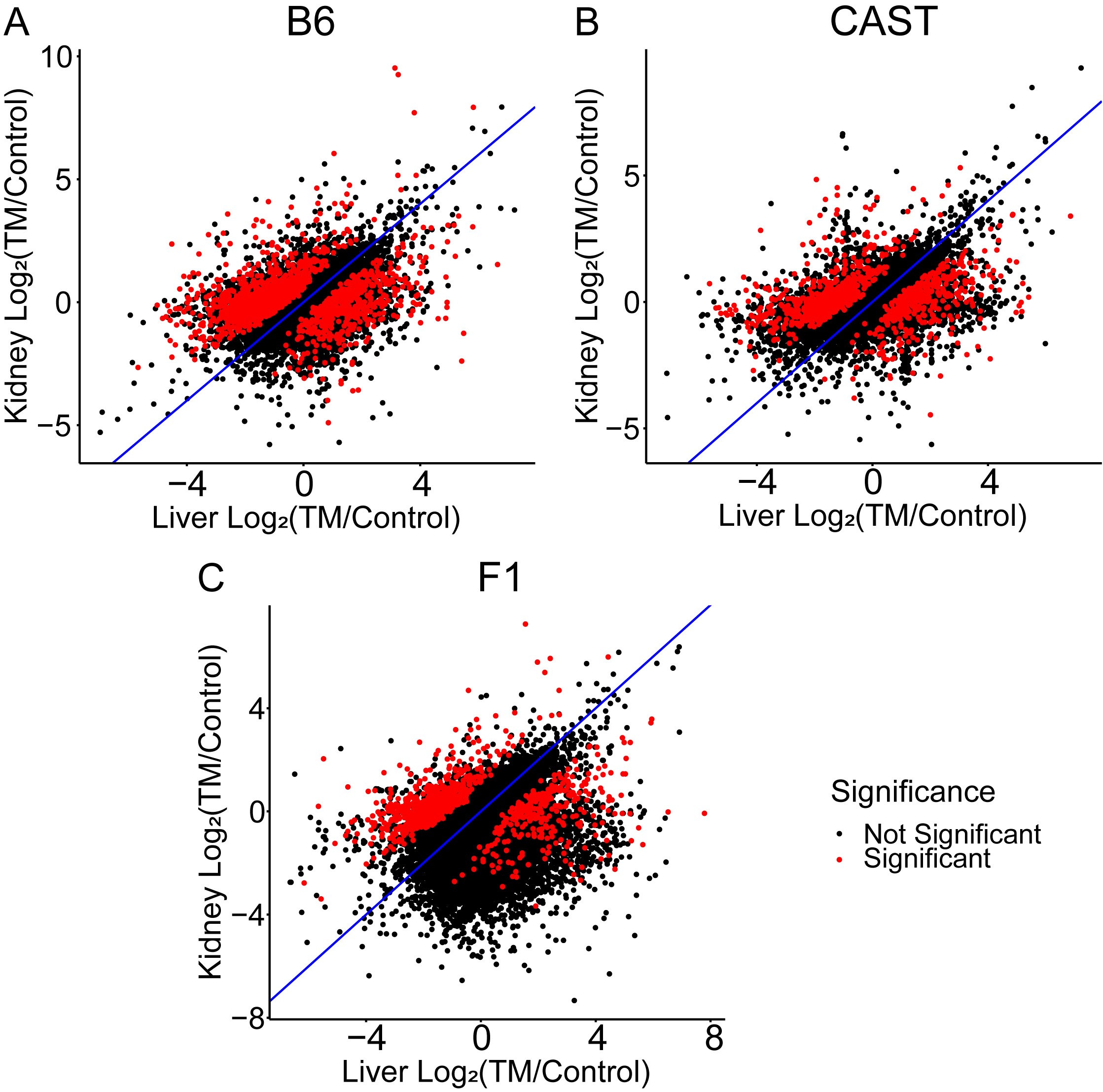

Supplement: jkac104_Supplementary_Figure_S5 [file jkac104_supplementary_figure_s5.jpeg]

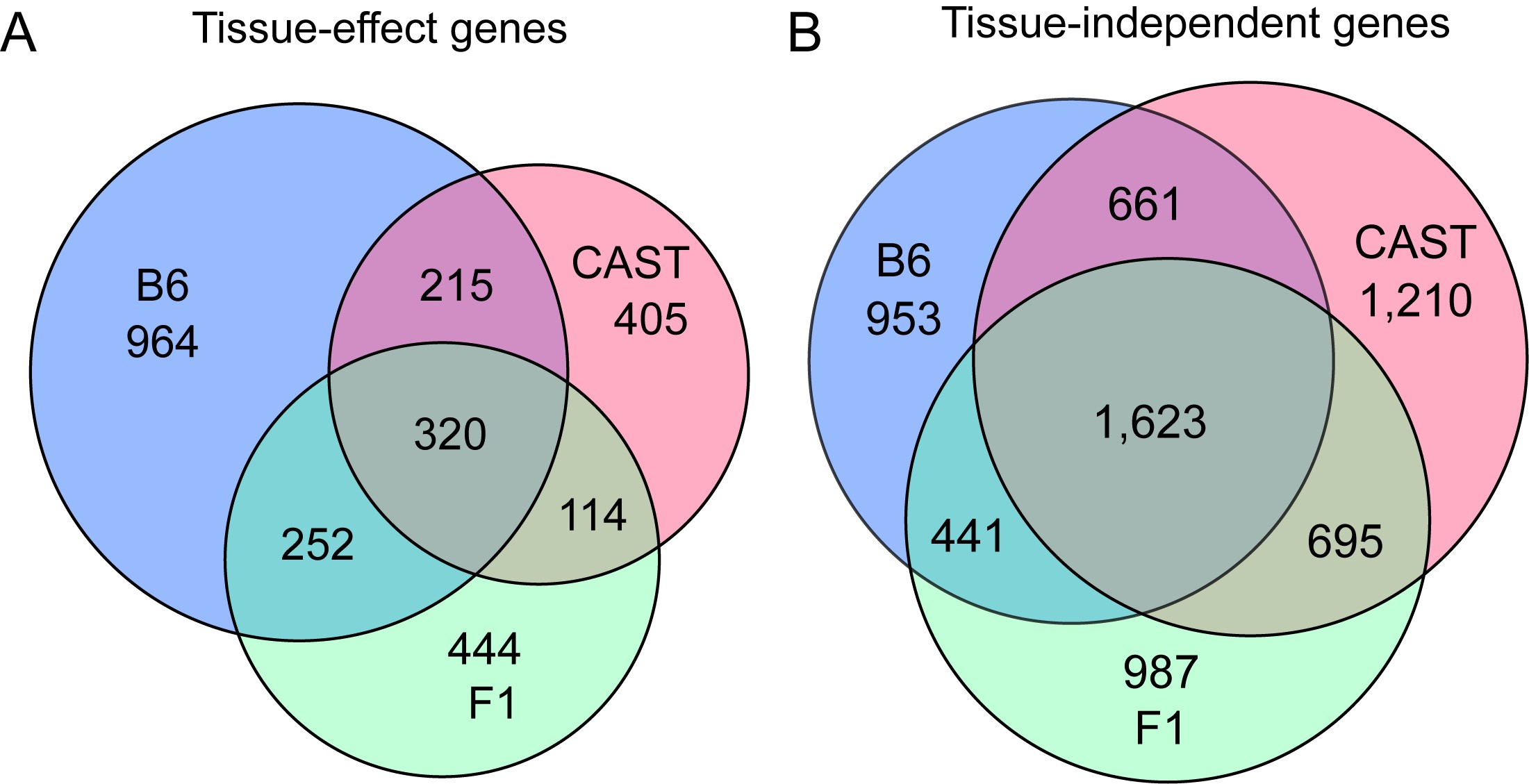

Supplement: jkac104_Supplementary_Figure_S6 [file jkac104_supplementary_figure_s6.jpeg]

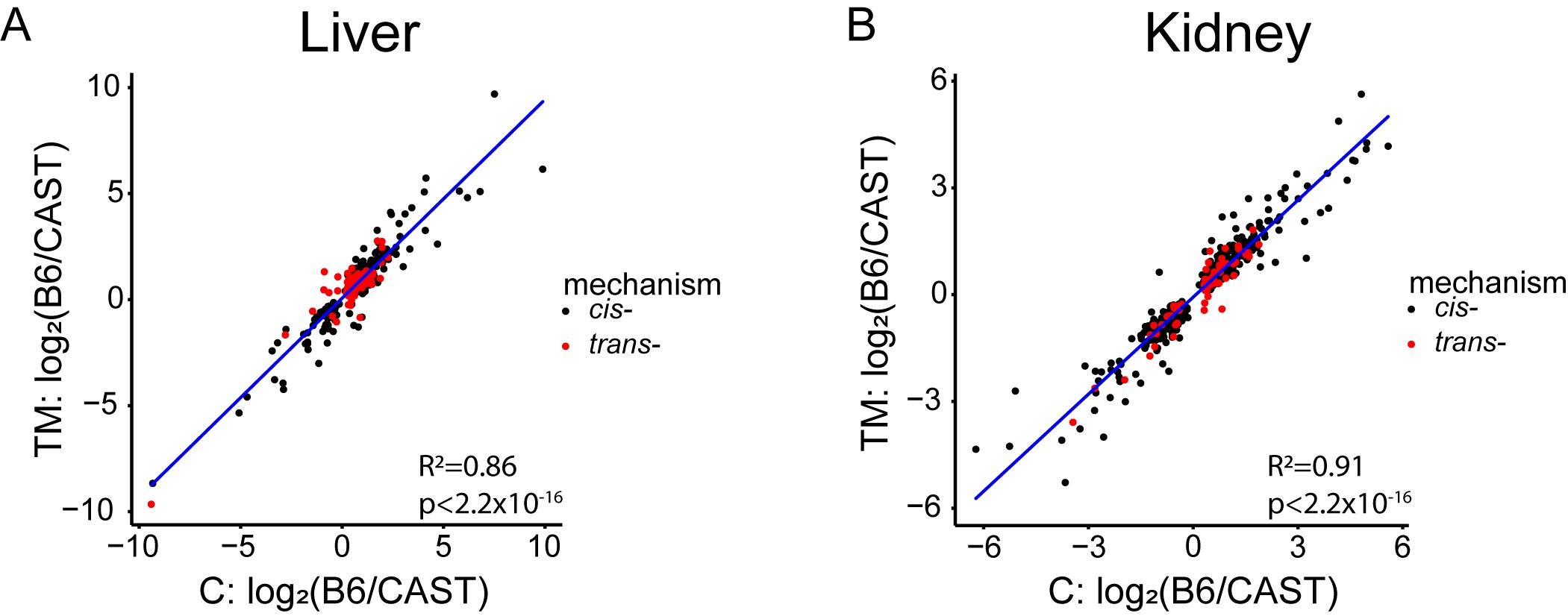

Supplement: jkac104_Supplementary_Figure_S7 [file jkac104_supplementary_figure_s7.jpeg]

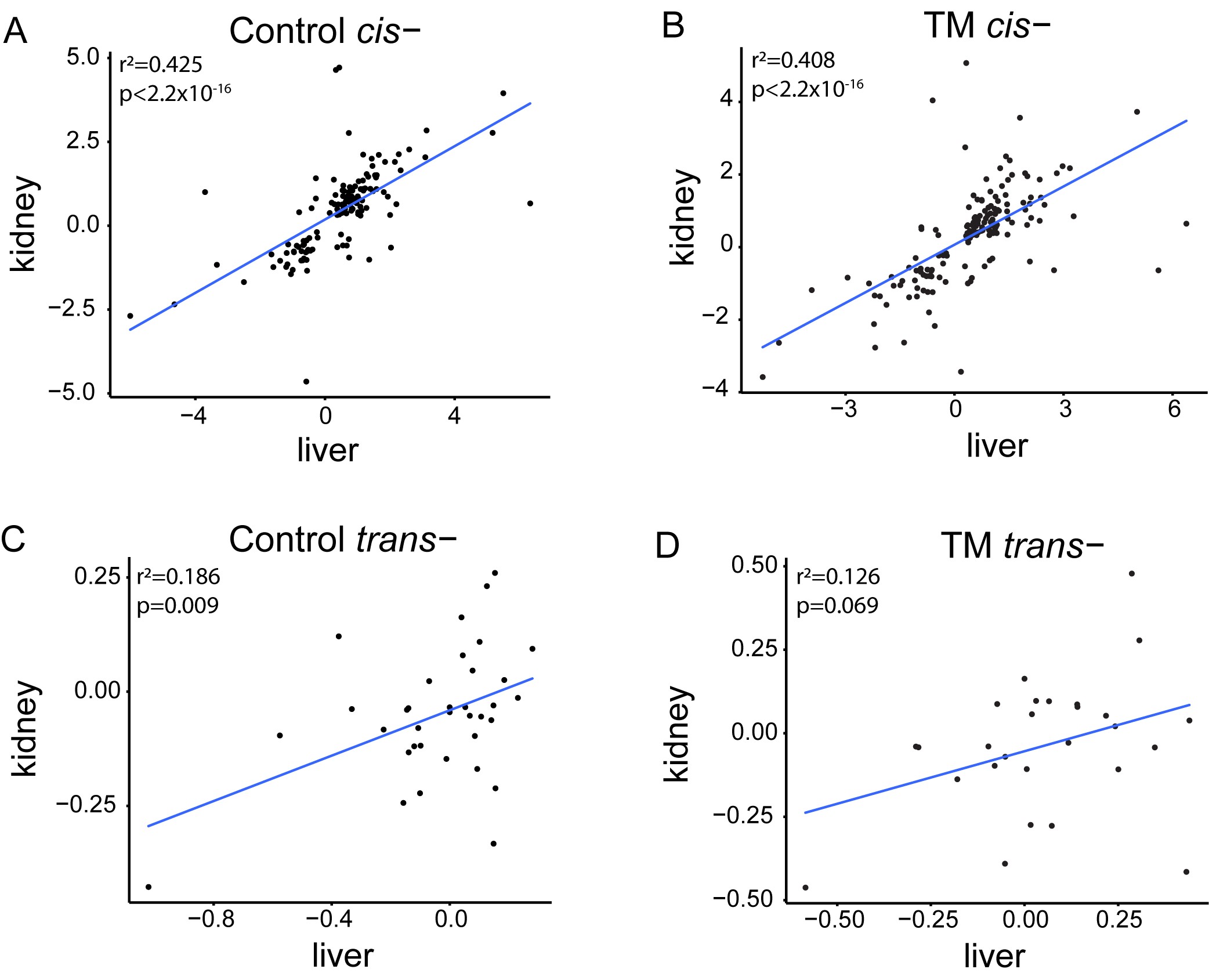

Supplement: jkac104_Supplementary_Figure_S8 [file jkac104_supplementary_figure_s8.jpeg]

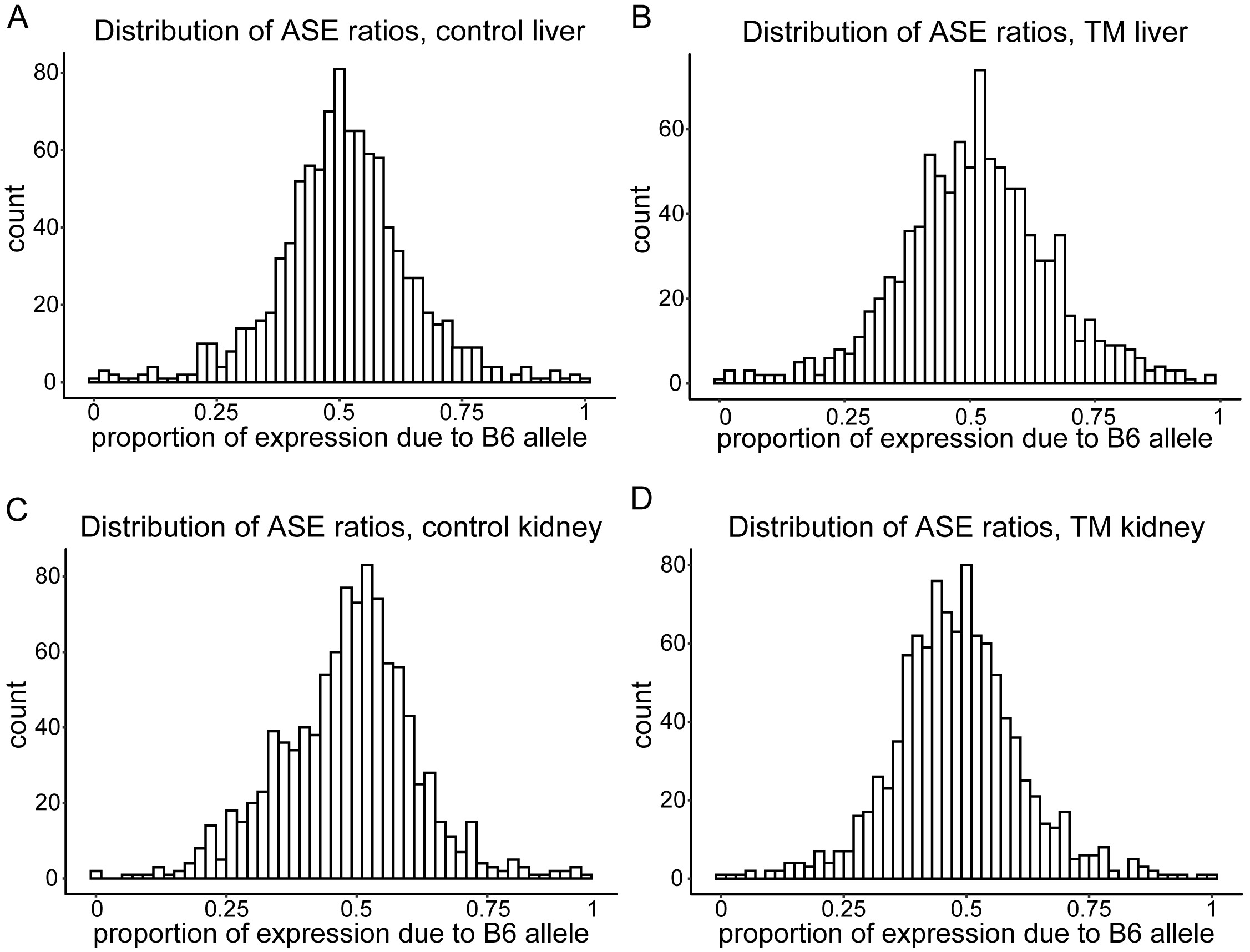

Supplement: jkac104_Supplementary_Figure_S9 [file jkac104_supplementary_figure_s9.jpeg]

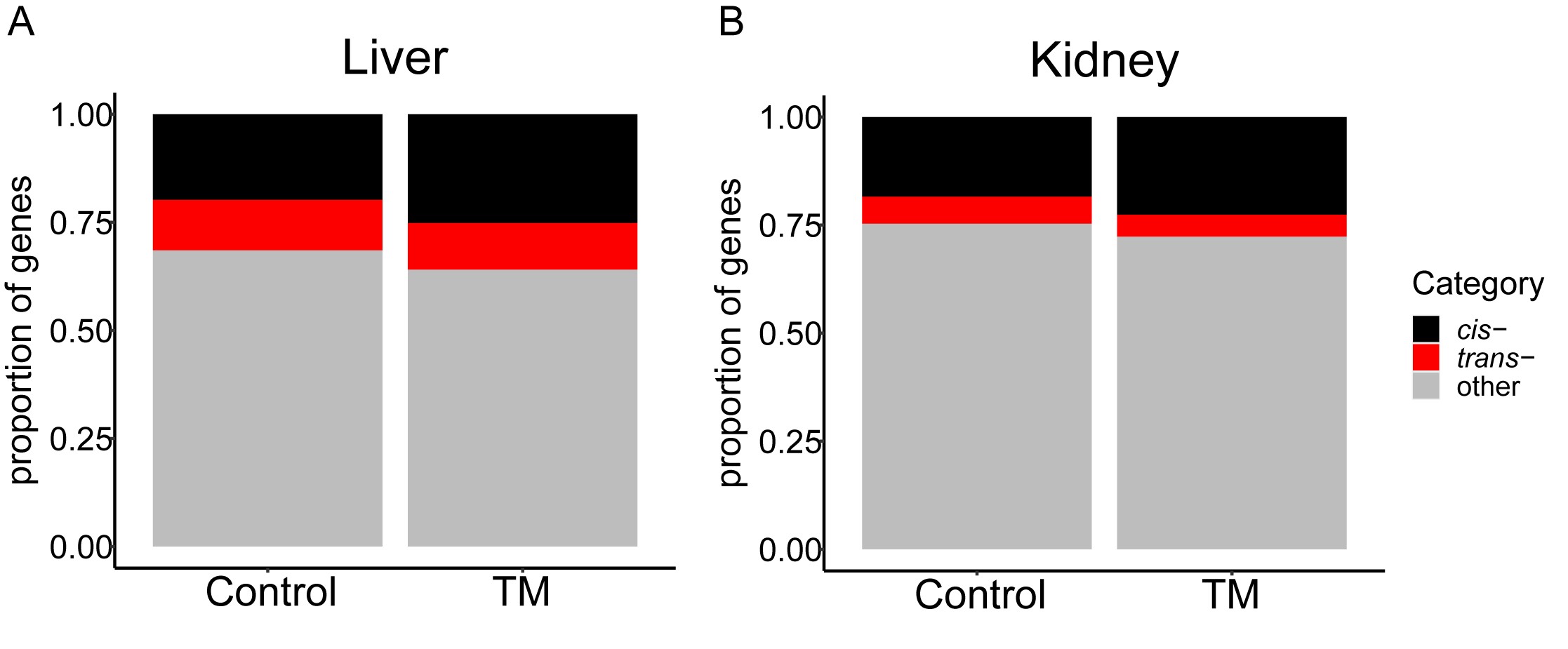

Supplement: jkac104_Supplementary_Figure_S10 [file jkac104_supplementary_figure_s10.jpeg]

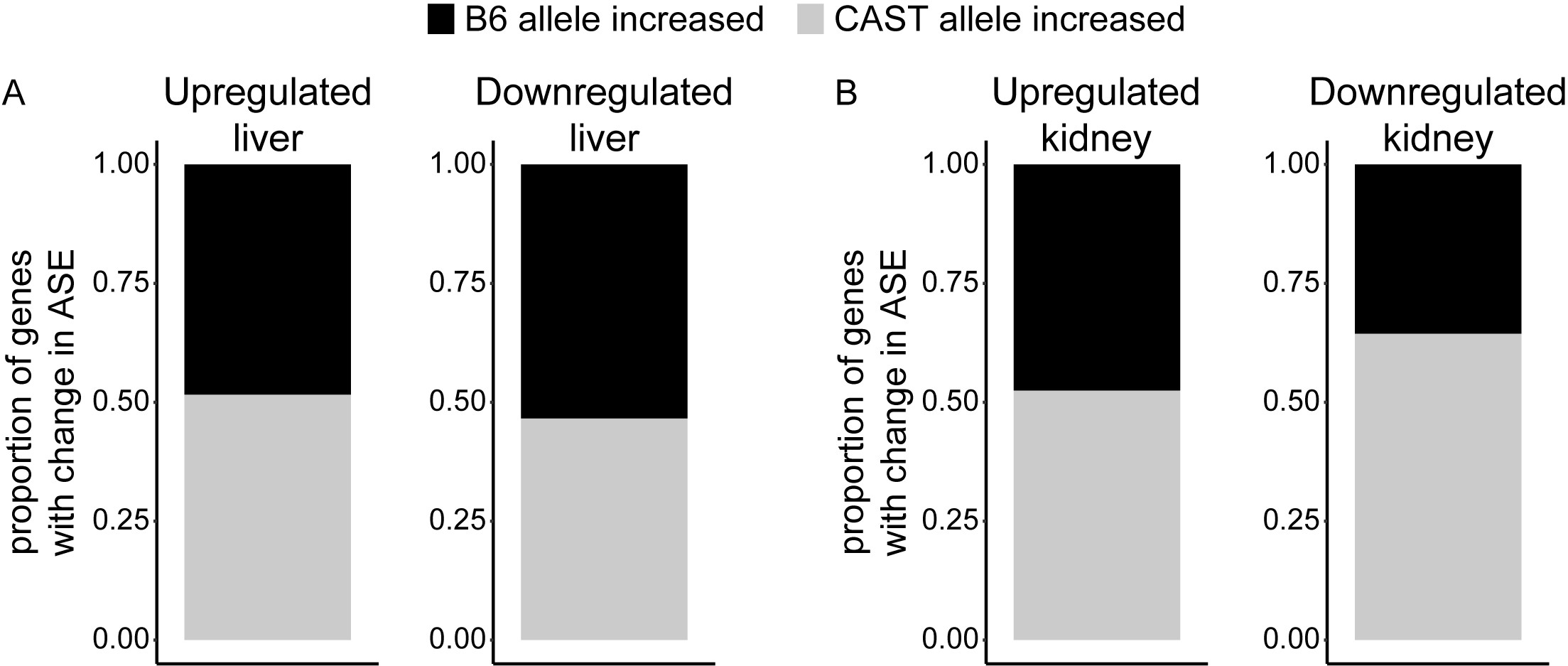

Supplement: jkac104_Supplementary_Figure_S11 [file jkac104_supplementary_figure_s11.jpeg]

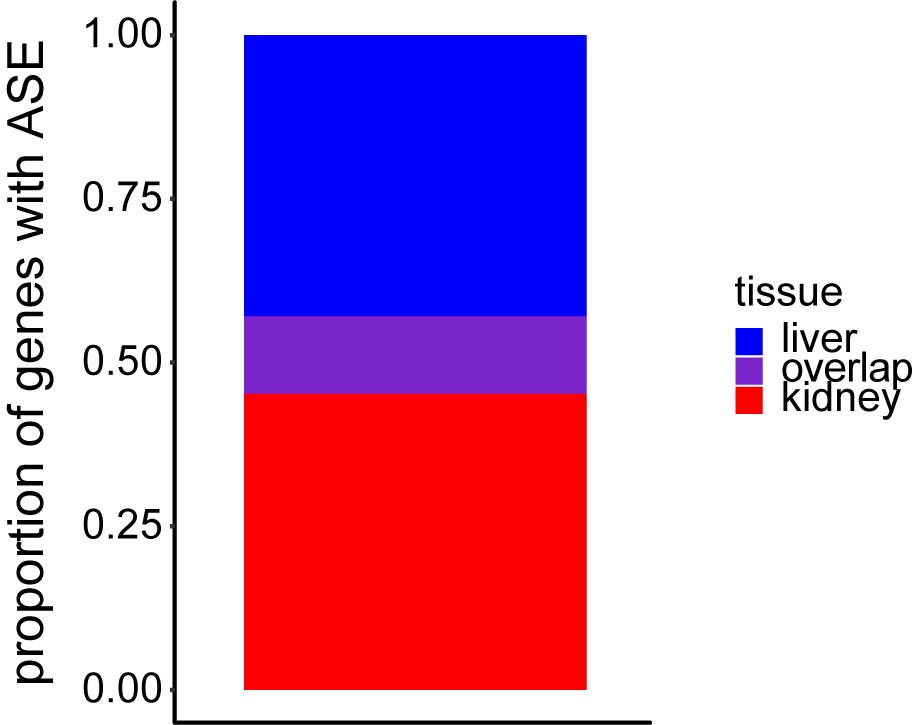

Supplement: jkac104_Supplementary_Figure_S12 [file jkac104_supplementary_figure_s12.jpeg]
